# Multi-analyte approach combining cfDNA sequencing and protein testing for early ovarian cancer detection

**DOI:** 10.1016/j.isci.2025.112617

**Published:** 2025-05-08

**Authors:** Fenfen Wang, Lingfang Wang, Ziyu Xing, Lingjia Lu, Zhuoqun Lin, Senmi Qian, Tao Zhu, Zhuyan Shao, Lingjun Zhao, Jie Dong, Fangfang Qian, Yang Li, Xiaojing Chen, Siqi Yang, Xiaodong Cheng

**Affiliations:** 1 Department of Gynecologic Oncology, Women’s Hospital, School of Medicine, Zhejiang University, Hangzhou, China; 2 Zhejiang Key Laboratory of Precision Diagnosis and Therapy for Major Gynecological Diseases, Women’s Hospital, Zhejiang University School of Medicine, Hangzhou, Zhejiang, China; 3 Zhejiang Provincial Clinical Research Center for Obstetrics and Gynecology, Hangzhou, China; 4 Zhejiang Provincial Key Laboratory of Traditional Chinese Medicine for Reproductive Health Research, Hangzhou, China; 5 Zhejiang Cancer Hospital, Hangzhou Institute of Medicine (HIM), Chinese Academy of Sciences, Hangzhou, China; 6 Ningbo Women and Children’s Hospital, Ningbo, China; 7 Huzhou Maternity & Child Health Care Hospital, Huzhou, China; 8 Department of Gynecology, Hangzhou Third People’s Hospital, Hangzhou, China

**Keywords:** Health sciences, Medicine, Medical specialty, Internal medicine, Oncology

## Abstract

Non-invasive liquid biopsy is a promising strategy for ovarian cancer (OC) detection. We evaluated the feasibility and efficacy of a multi-analyte approach combining circulating tumor DNA (ctDNA) detection with protein biomarkers for early OC detection. At 95% specificity, CA125 and ctDNA alone exhibited an overall sensitivity of 79.0% and 58.7%, respectively; however, when CA125 was combined with ctDNA, sensitivity reached 85.5%. Integrating CA125 and human epididymis protein-4 (HE4) in the risk of ovarian malignancy algorithm (ROMA) index, yielded a sensitivity of 86.2%. When four additional proteins (HE4, cancer antigen 19-9, prolactin, and interleukin-6) were added to CA125 and ctDNA in the EarlySEEK model, sensitivity increased to 94.2%. Meanwhile, the EarlySEEK model was not affected by menopausal status, and outperformed CA125 in distinguishing benign and malignant ovarian tumors. These findings demonstrate that EarlySEEK could effectively identify OC at early stage when they are more likely to be curable.

## Highlights

• We developed EarlySEEK by combining ctDNA and protein biomarkers for early detection of OC
• EarlySEEK model had a sensitivity of 93.2% in early detection of OC
• The performance of EarlySEEK model was unaffected by age or menopausal status
• EarlySEEK model can be used to differentiating the benign and malignant adnexal cysts

## Graphical abstract

**Figure:**
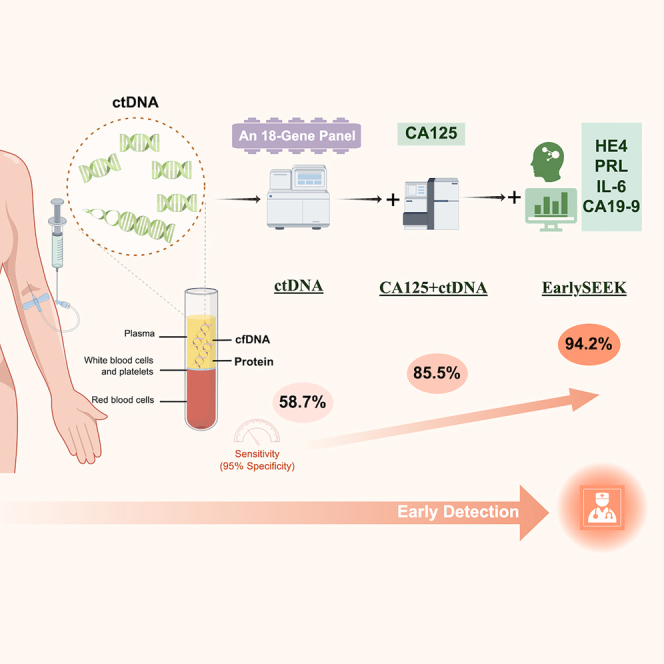

## Introduction

Ovarian cancer (OC) is the leading cause of death in women with gynecological malignancies. In China, 55,342 new OC cases and 37,519 OC-related deaths were reported in 2020.^1^ Epithelial OC (EOC) accounts for approximately 90% of all OC cases, and over 70% of all EOC cases are diagnosed at late stages.^2^ The 5-year overall survival (OS) in patients with OC is < 40% at the advanced stage, but > 90% at the early stage.^2^^,^^3^^,^^4^ Thus, early detection of OC is critical for effective treatment and better survival; however, there are currently no reliable methods or biomarkers for early OC detection.

Blood tests for the detection of cancer antigen-125 (CA125) and human epididymis protein-4 (HE4), as well as imaging approaches, such as computed tomography, positron emission tomography, magnetic resonance imaging, and transvaginal ultrasound (TVS), are commonly used for early OC detection.^5^^,^^6^ TVS and CA125 detection are simple and cost-effective methods for early OC detection. However, CA125 levels are affected by various factors, such as endometriosis and abscesses, and its levels are undetectable in up to 50% of patients with stage I OC; therefore, it is not a reliable indicator for early OC detection.^7^^,^^8^ Furthermore, prospective studies have shown that CA125 alone or in combination with TVS can only be used to identify 30%–45% of early-stage OC cases.^9^^,^^10^^,^^11^ Although HE4 has been reported to be more reliable for OC detection than CA125, its expression in patients with OC varies significantly with age and histological subtype; therefore, its usefulness for OC detection is limited.^12^^,^^13^^,^^14^^,^^15^^,^^16^

Recent advances in liquid biopsy detection techniques based on the analysis of multiple protein biomarkers in serum or plasma, circulating tumor DNA (ctDNA) in plasma, uterine fluid, and exosomes have improved the sensitivity of early detection methods.^17^^,^^18^^,^^19^^,^^20^ However, studies that have incorporated CA125 and HE4 with other protein biomarkers such as cancer antigen 19-9 (CA19-9), carcinoembryonic antigen (CEA), and alpha-fetoprotein (AFP) have certain limitations.^21^^,^^22^^,^^23^ First, the majority of these studies included limited sample size of patients with early-stage OC. Second, several of these biomarkers could only be used to detect a few OC cases missed by CA125. Third, the combination models used in these studies were not externally verified.^6^

Recent studies have shown that low-prevalence mutations in cell-free DNA (cfDNA) can be detected with high sensitivity, allowing for early cancer detection.^24^^,^^25^ However, mutations in ctDNA have been identified in 29.6% of patients with benign ovarian lesions involving gynecological organs.^24^^,^^25^^,^^26^ This non-invasive and repeatable detection method has significantly improved the diagnosis of multiple cancers, including OC. The PapSEEK model yielded a 43% OC detection rate using ctDNA alone.^27^ Protein testing combined with ctDNA testing has been used in studies seeking to improve the detection of lung, liver, and pancreatic cancer.^28^^,^^29^^,^^30^ The CancerSEEK model, which integrates ctDNA and eight protein biomarkers in a multi-analyte blood test, demonstrated an overall OC detection sensitivity and specificity of 98% and > 99%, respectively.^31^ These studies suggest that combining ctDNA with protein biomarkers may increase diagnostic sensitivity for early-stage OC. However, the OC cohort in the CancerSEEK study included only 54 patients, with only 24% of them having stage I and II OC. Furthermore, the workflow for ctDNA mutation detection is complicated.^6^^,^^31^ Thus, there is a need to identify optimum biomarker combinations with efficient workflow to improve OC detection, particularly in patients with early-stage OC.

In this study, we aimed to develop predictive models that combine ctDNA detection with a variety of protein biomarkers and to assess their performance in early detection and identification of benign and malignant ovarian tumors.

## Results

### Characteristics of the participants

**Figure 1:**
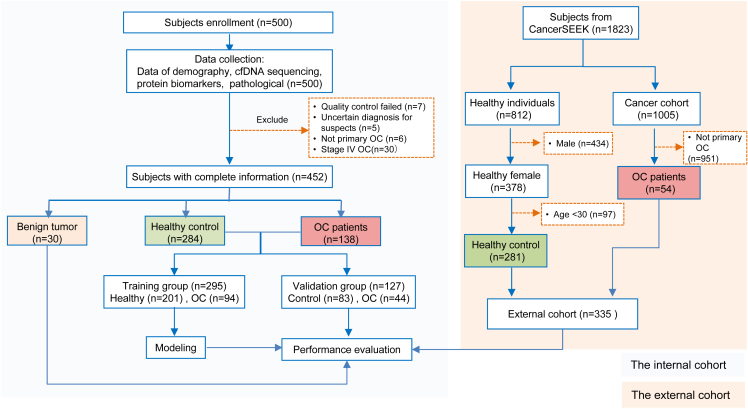
Workflow of the study In the internal cohort, 500 individuals were enrolled into the study, and after data collection, 7 individuals were excluded because of quality control failed and 5 individuals were excluded because of the uncertain diagnosis, 6 individuals were excluded because of the diagnosis of not primary OC and 30 patients were excluded for being diagnosed with stage IV OC. At last, 452 individuals were included in this study. The training group (*n* = 295) was used to develop logistic models for early detection of ovarian cancer (OC). The validation group (*n* = 127) was used to evaluate the performance of the models. Dataset extracted from CancerSEEK was used as external validation cohort (*n* = 335). A mixture of benign tumor (*n* = 30) and OC cases (*n* = 138) was used to assess the effectiveness of the established model in distinguishing benign from malignant tumors. OC, ovarian cancer; cfDNA, cell-free DNA.

Figure 1 shows the flowchart of the study design. A total of 452 participants were included in the internal cohort, including 138 patients with OC, with 87.7% (121/138) of them having EOC, and 314 controls consisting of 30 patients with benign tumors and 284 healthy individuals (Tables 1 and S1). The external cohort included 335 subjects from the CancerSEEK study, consisting of 54 patients with OC and 281 healthy women. The characteristics of the participants are shown in Tables 1 and S1. The average ages of the healthy individuals, patients with benign tumors, and patients in the OC cohort were 58.7 ± 6.1, 48.6 ± 13.1, and 55.1 ± 11.7 years, respectively. Median CA125 level was 9.8 U/mL (interquartile range [IQR]: 7.8–15.6) in the healthy individual group, 21.2 U/mL (IQR: 13.1–86.2) in the benign tumor group, and 261 U/mL (IQR: 69.2–634.1) in the OC cohort. Patients with stage I, II, and III OC accounted for 27.5%, 19.6%, and 52.9% of the total number of patients with OC, respectively. Healthy individuals and patients with OC were randomly assigned to the training and validation sets at a 7:3 ratio. There were 295 participants in the training cohort (201 healthy, 94 OC) and 127 in the validation cohort (83 healthy, 44 OC). Age (t test, *p* > 0.5), CA125 levels (Mann-Whitney U test, *p* = 0.398), histological types (Fisher’s exact test, *p* = 0.54), and tumor stages (Chi-squared test, *p* = 0.43) were comparable between the training and validation groups (Table 1). Patients’ histological type varied with tumor stage (Fisher’s exact test, *p <* 0.001, Table S2), with the clear cell carcinoma (CCC) and mucinous carcinoma (MC) histological subtypes being more frequently diagnosed at earlier stages, and the high-grade serous carcinoma (HGSC) histological subtype being more frequently detected in stage III (Table S2).

**Table 1:** Characteristics and demographics of the participants of the study

Variables	All subjects (*n* = 452)			Healthy cohort (*n* = 284)			OC cohort (*n* = 138)		
	Healthy (*n* = 284)	Benign tumor (*n* = 30)	OC (*n* = 138)	Training (*n* = 201)	Validation (*n* = 83)	*p* values	Training (*n* = 94)	Validation (*n* = 44)	*p* values
Age (Years)									
Mean (SD)	58.7 (6.1)	48.6 (13.1)	55.1 (11.7)	53.4 (9.7)	52.7 (10.2)	0.585	54.8(11.2)	55.8 (13.0)	0.665
<50 [n (%)]	95 (33.5)	15 (50.0)	35 (25.4)	62 (30.8)	33 (39.8)	0.148	24 (25.5)	11 (25.0)	0.947
≥50 [n (%)]	189 (66.6)	15 (50.0)	103 (74.6)	139 (69.2)	50 (60.2)		70 (74.5)	33 (75.0)	
CA125 level (U/ml)									
Median (IQR)	9.8 (7.8–15.6)	21.2 (13.1–86.2)	261 (69.2–634.1)	9.5 (7.7–15.3)	10.5 (8.0–16.2)	0.345	247.9 (43.7–616.2)	278.2 (135.0–694.4)	0.398
FIGO stage	n (%)								0.434
I	–	–	38 (27.5)	–	–	–	29 (30.9)	9 (20.4)	
II	–	–	27 (19.6)	–	–	–	18 (19.1)	9 (20.4)	
III	–	–	73 (52.9)	–	–	–	47 (50.0)	26 (59.1)	
Tumor subtype	n (%)								
Non-EOC	–	–	17 (12.3)	–	–	–	12 (12.8)	5 (11.4)	0.815
EOC	–	–	121 (87.7)	–	–	–	82 (87.2)	39 (88.6)	
Serous carcinoma	–	–	93 (67.4)	–	–	–	62 (66.0)	31 (70.5)	0.543
Mucinous carcinoma	–	–	5 (3.6)	–	–	–	4 (4.3)	1 (2.3)	
Clear cell carcinoma	–	–	15 (10.9)	–	–	–	12 (12.8)	3 (6.8)	
Endometrioid carcinoma	–	–	8 (5.8)	–	–	–	4 (4.3)	4 (9.1)	

OC, ovarian cancer; SD, standard deviation; CA125, cancer antigen-125; IQR, interquartile range; FIGO, International Federation of Gynecology and Obstetrics; EOC, epithelial ovarian carcinoma.

### ctDNA features of the participants

**Figure 2:**
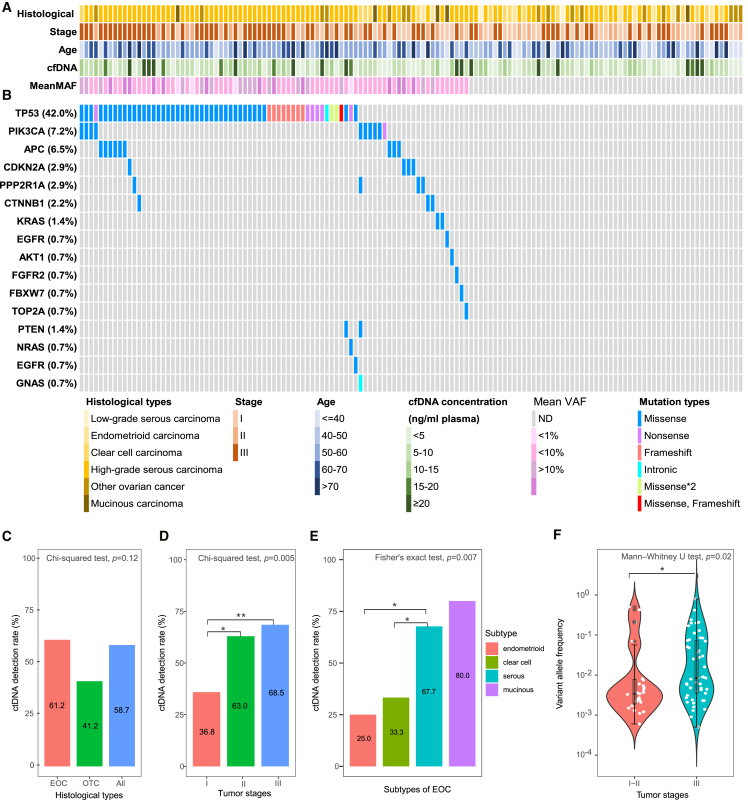
Circulating tumor DNA detection in 138 patients with OC (A) Clinical features, cell-free DNA (cfDNA) concentrations, and mean variant allele frequencies (VAFs) of the 138 patients with ovarian cancer (OC) included in this study. Each column represents one patient with OC. (B) Circulating tumor DNA (ctDNA) mutational landscapes of the 138 patients with OC included in this study. Each column represents one OC patient, and different color blocks represent different mutational statuses and mutation types. (C) Comparison of plasma ctDNA detection rate between patients with epithelial ovarian cancer (EOC) (*n* = 121) and those with other types of OC (OTC) (*n* = 17). (D) ctDNA detection rate in patients with OC stratified by tumor stage. The number of patients in stage I, II, and III were 38, 27, and 73 patients, respectively. (E) ctDNA detection rate stratified by histological subtype in 121 patients with EOC, including 15 patients with clear cell carcinoma, 8 patients with endometrioid carcinoma, 5 patients with mucinous carcinoma, and 93 patients with serous carcinoma. (F) Violin plot showing the variant allele frequency (VAF) distribution for ctDNA variants in patients with stage I and II (*n* = 65) and stage III (*n* = 73) OC. Each dot represents one variant. cfDNA: cell-free DNA; VAF: variant allele frequency; ND: not detected; EOC: epithelial OC; OTC: other types of OC; ∗*p* < 0.05; ∗∗*p* < 0.01; ∗∗∗*p* < 0.001. Groups with statistical differences are labeled.

The clinical characteristics and ctDNA mutational landscapes of patients with OC are shown in Figures 2A and 2B. In the OC cohort, the three most frequently mutated genes were *TP53* (42.0%), *PIK3CA* (7.2%), and *APC* (6.5%). The detection rates of these mutations were not significantly different between EOC and non-EOC patients (Chi-squared test, *p* ≥ 0.05, Table S3). We used whole exon sequencing to sequence paired OC tissue samples from 32 ctDNA mutation-positive patients and found *TP53*, *PIK3CA*, and *APC* were also the most frequently mutated genes in the tumor biopsies, and the concordance rate between ctDNA and tumor tissues was 68.8% (Table S4), which was similar to that reported in previous studies.^32^

The overall ctDNA detection rate was 5.3% (15/284), 23.3% (7/30), and 58.7% (81/138), respectively, in healthy individuals, patients with benign tumors and patients with OC (Chi-squared test, *p* < 0.001; Table S1 and S5). The median VAF for the ctDNA-positive OC, benign tumor, and healthy individual groups was 0.60% (IQR: 0.24–5.08%), 0.19% (IQR: 0.17–0.21%), and 0.14% (IQR: 0.08–0.21%), respectively, with significant differences observed (Kruskal-Wallis test, *p* < 0.001; Table S5). The OC, benign tumor, and healthy individual groups exhibited median cfDNA concentrations of 7.22 (IQR: 4.86–10.74), 6.24 (IQR: 4.52–9.05), and 6.14 (IQR: 3.54–9.74), respectively, with no significant differences observed (Kruskal-Wallis test, *p* = 0.163; Table S5).

The ctDNA detection rate was 58.7% (81/138) in all patients with OC, 61.2% (74/121) in patients with EOC, and 41.2% (7/17) in non-EOC patients, and there was no significant difference in ctDNA detection rate between the EOC and non-EOC groups (Chi-squared test, *p* = 0.12, Figure 2C). The ctDNA detection rates for stages I–III OC were 36.8% (14/38), 63.0% (17/27), and 68.5% (50/73), respectively. The detection rate for stage I OC was significantly lower than that for stage II (Chi-squared test, *p =* 0.038) and III (Chi-squared test, *p* = 0.001) OC (Figures 2D; Table S3). The detection rates of the endometrioid carcinoma (EC), CCC, serous carcinoma (SC), and MC in the EOC subgroup were 25.0% (2/8), 33.3% (5/15), 67.7% (63/93), and 80.0% (4/5), respectively (Figures 2E; Table S3). The detection rate of the SC histological subtype differed significantly from that of the CCC (Chi-squared test, *p* = 0.01) and EC (Fisher’s exact test, *p* = 0.02) histological subtypes. However, the ctDNA detection rate for the other EOC subtypes did not differ significantly (Figure 2E). The median variant allele frequency (VAF) in ctDNA-positive patients with stage I/II and stage III OC was 0.34% (IQR: 0.19–0.78%) and 0.84% (IQR: 0.36–7.23%), respectively, with a significant difference observed between the two groups (Mann-Whitney U test, *p* = 0.02; Figure 2F).

### Simultaneous analysis of plasma samples for CA125 and ctDNA

**Figure 3:**
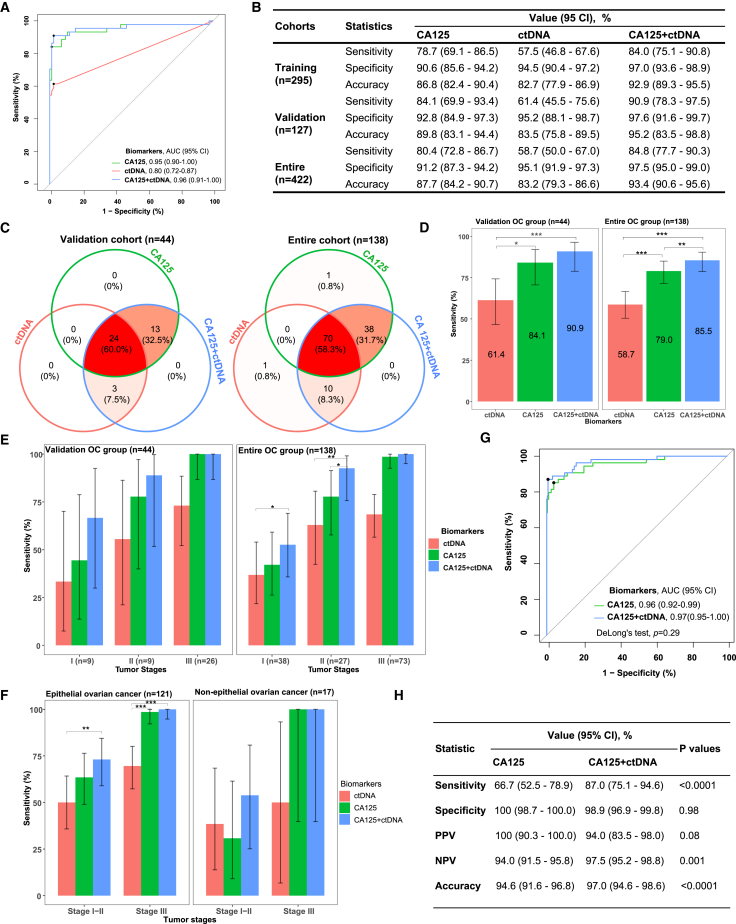
Performance comparison between CA125 alone, ctDNA alone, and the combination test (A) Receiver operator characteristic (ROC) curves for CA125 alone, ctDNA alone, and the combination test using the validation dataset (*n* = 127). (B) Comparison of sensitivity, specificity, and accuracy between the different biomarkers in the training, validation and the entire dataset. The cutoff value for CA125 was 35 U/mL; the cutoff values for the ctDNA and CA125+ctDNA models were defined using the Youden Index. (C) Venn diagram showing the number and proportion of patients with OC identified using the different biomarkers at 95% specificity in the validation and entire cohort. (D) Overall sensitivities for CA125 alone, ctDNA alone, and the combination test at 95% specificity in the validation and entire cohort. (E) Sensitivity of different biomarkers stratified by tumor stage at 95% specificity in the validation and entire cohort. (F) Sensitivity of different indicators stratified by tumor stage at 95% specificity in patients with epithelial OC and non-epithelial OC. (G) ROC curves for CA125 alone and CA125+ctDNA in the CancerSEEK external validation cohort. (H) Comparison of sensitivity, specificity, and accuracy between the two biomarkers in the external validation cohort. The black lines indicate the 95% confidence intervals for sensitivity. AUC: area under the curve; CI: confidence interval; ∗*p* < 0.05; ∗∗*p* < 0.01; ∗∗∗*p* < 0.001. Groups with statistical differences are labeled.

CA125 is routinely used for early OC diagnosis and monitoring. Thus, we sought to determine whether combining ctDNA detection with CA125 would improve diagnostic sensitivity with respect to that obtained when the two biomarkers are used alone. We developed a model using data from the training group and evaluated its performance using data from the validation group. Age (Kruskal-Wallis test, *p* = 0.33) and cfDNA concentration (Kruskal-Wallis test, *p* = 0.06) did not significantly differ between healthy individuals and patients with OC (Figure S1A). Among cfDNA or ctDNA features, ctDNA VAF showed the highest area-under-the-curve (AUC) value of 0.80 (95% confidence interval (CI): 0.72–0.87) in the validation group, which was higher than that of cfDNA concentration (DeLong’s test, *p* = 0.0002), and was similar to that of the number of ctDNA variants (DeLong’s test, *p* = 0.07) (Figure S1B). In the validation set, the combined test (CA125+ctDNA) showed the highest AUC value of 0.96 (95% CI: 0.91–1.00), which was significantly higher than that for ctDNA alone (DeLong’s test, *p* = 0.002); however, the AUC for the combination test was not significantly different from that for CA125 alone (DeLong’s test, *p* = 0.33) (Figure 3A). Using the Youden index in the validation set, the sensitivity of the combination test was found to reach 90.9% (95% CI: 78.3%–97.5%) at 97.6% (95% CI: 91.6%–99.7%) specificity and 95.2% (95% CI: 83.5%–98.8%) accuracy, CA125 detection alone showed a sensitivity of 84.1% (95% CI: 69.9%–93.4%) at 92.8% specificity (95% CI: 84.9%–97.3%) and 89.8% (95% CI: 83.1%–94.4%) accuracy, ctDNA detection alone showed a sensitivity of 61.4% (95% CI: 45.5%–75.6%) at 95.2% (95% CI: 88.1%–98.7%) specificity and 83.5% (95% CI: 75.8%–89.5%) accuracy (Figures 3B; Table S6). CA125+ctDNA detection exhibited higher accuracy than ctDNA and CA125 detection alone (Figures 3B; Table S6). Throughout the cohort, CA125+ctDNA and CA125 alone exhibited comparable sensitivities (McNemar’s test, *p* = 0.06), while CA125+ctDNA significantly outperformed CA125 alone in terms of specificity (McNemar’s test, *p <* 0.0001), predictive positive value (PPV) (Chi-squared test, *p* < 0.0001), predictive negative value (NPV) (Chi-squared test, *p =* 0.02), and accuracy (McNemar’s test, *p <* 0.0001) (Table S6). To further explore the performance of these models in early detection of OC, we divided OC patients in the validation set into stage I + II and stage III subgroups. The AUC values were 0.88,0.69, and 0.90, respectively, in CA125 alone, ctDNA alone, and CA125+ctDNA in stage I + II subgroup.CA125 and ctDNA alone exhibited a low accuracy of 88.1% and 85.1%, respectively; however, when CA125 was combined with ctDNA, accuracy reached 93.1% in stage I + II subgroup. As for stage III subgroup, we found there were comparable AUC value, sensitivities, specificity and accuracy with CA125 alone and CA125+ctDNA (Figure S2).

High specificity was observed with the CA125+ctDNA model and with ctDNA detection alone (Figure 3B). Test specificity was adjusted to 95% to compare the sensitivities of different biomarkers. Figure 3C shows the number and proportion of patients with OC identified using CA125 alone, ctDNA alone, and the CA125+ctDNA combination test in the validation and OC groups. We found that 9.1% (4/44) of the cases in the validation set and 13.0% (18/138) of the patients in the entire OC group were not detected by any biomarker, and all three indicators could identify OC in nearly 60% of the patients with OC (Figure 3C). In addition, the CA125+ctDNA model could detect three cases and ten patients missed by CA125 in the validation and OC groups, respectively (Figure 3C). The sensitivity of ctDNA alone was only 61.4% (95% CI: 46.6%–74.3%) at 95% specificity; however, when CA125 was added to the model, this sensitivity increased to 90.9% (95% CI: 78.8%–96.4%) in the validation cohort (McNemar’s test, *p =* 0.0003, Figure 3D), suggesting that the CA125+ctDNA model outperformed CA125 and ctDNA alone in early OC detection. Furthermore, in the overall stage II subgroup, the sensitivity of the CA125+ctDNA model was higher than that of ctDNA alone and CA125 alone in the entire OC group, with statistical significance (Figure 3E). Moreover, the detection rate of early-stage OC (stage I and II) appeared to be higher in EOC groups than in non-EOC group for almost all the indicators; however, only CA125 showed a statistically significant difference (Chi-squared test, *p =* 0.03). There was no difference in sensitivity between CA125, ctDNA, and CA125+ctDNA in the non-EOC group (Figure 3F).

We validated the CA125+ctDNA model using external data from 335 participants of the CancerSEEK study (Table S7). In this dataset, the CA125+ctDNA model had a similar AUC value to CA125 alone (DeLong’s test, *p =* 0.29, Figure 3G); the overall performance of CA125+ctDNA was also significantly higher than that of CA125 in terms of sensitivity, NPV, and accuracy (Figure 3H). The sensitivity (Chi-squared test, *p =* 0.69), specificity (Chi-squared test, *p =* 0.34), and accuracy (Chi-squared test, *p =* 0.69) of the CA125+ctDNA model was similar to that of our dataset (Figures 3B and 3H).

### Increasing sensitivity by including additional protein biomarkers

Based on the findings of the CancerSEEK study^31^ and those of a previous study of ours,^33^ we speculated that integrating ctDNA detection with multiple protein biomarkers could improve the sensitivity of early detection for multiple cancers. Based on the results shown above (Figure 3), we discovered that, while CA125+ctDNA outperformed CA125 alone and ctDNA alone, the detection sensitivity of early-stage OC, particularly stage I OC still requires improvement. Thus, we attempted to integrate more protein biomarkers into our model to improve its performance. We included the protein biomarkers, HE4, CA19-9, CEA, and AFP, which are all commonly used in clinical cancer detection, as well as prolactin (PRL) and interleukin-6 (IL-6), which have been reported in previous studies.^22^^,^^23^^,^^31^^,^^34^^,^^35^^,^^36^^,^^37^^,^^38^ CEA levels did not differ between the OC and healthy individual groups (Kruskal-Wallis test, *p =* 0.21), whereas HE4, IL-6, PRL, and CA19-9 levels were higher in the OC group than in the healthy individual group (Figure S3A). Furthermore, traditional univariate and multivariate logistic regression (LR) analyses revealed CA125, HE4, PRL, and IL-6 levels as independent factors associated with OC detection (Table S8). Moreover, HE4 and IL-6 alone exhibited high AUC values of 0.91 (95% CI: 0.85–0.98) and 0.86 (95% CI: 0.78–0.94), respectively, in the validation cohort (Figure S3B). Thus it was difficult to decide which or how many factors to be further included in the CA125+ctDNA model through these traditional methods. To determine the optimal biomarker combination, we employed 5-fold cross-validation with three metrics in the Scikit-learn (sklearn) training cohort. A total of 8 variables, comprising six proteins (HE4, CEA, CA19-9, AFP, PRL, and IL-6), age, and cfDNA concentration, were randomly blended with CA125+ctDNA to generate 255 combination (Table S9). In addition, we attempted different combinations of the 10 factors (ctDNA, CA125, HE4, CEA, CA19-9, AFP, PRL, IL-6, age, cfDNA concentration), which yielded 1023 combinations (Table S9). Among these combinations, the combinations consisting of CA125, HE4, ctDNA detection, PRL, IL-6, and CA19-9, namely EarlySEEK, showed the best performance with the least number of variables; it exhibited a mean accuracy of 94.6%, a mean F1 score of 0.91, and a mean AUC value of 0.975 (Table S9).

**Figure 4:**
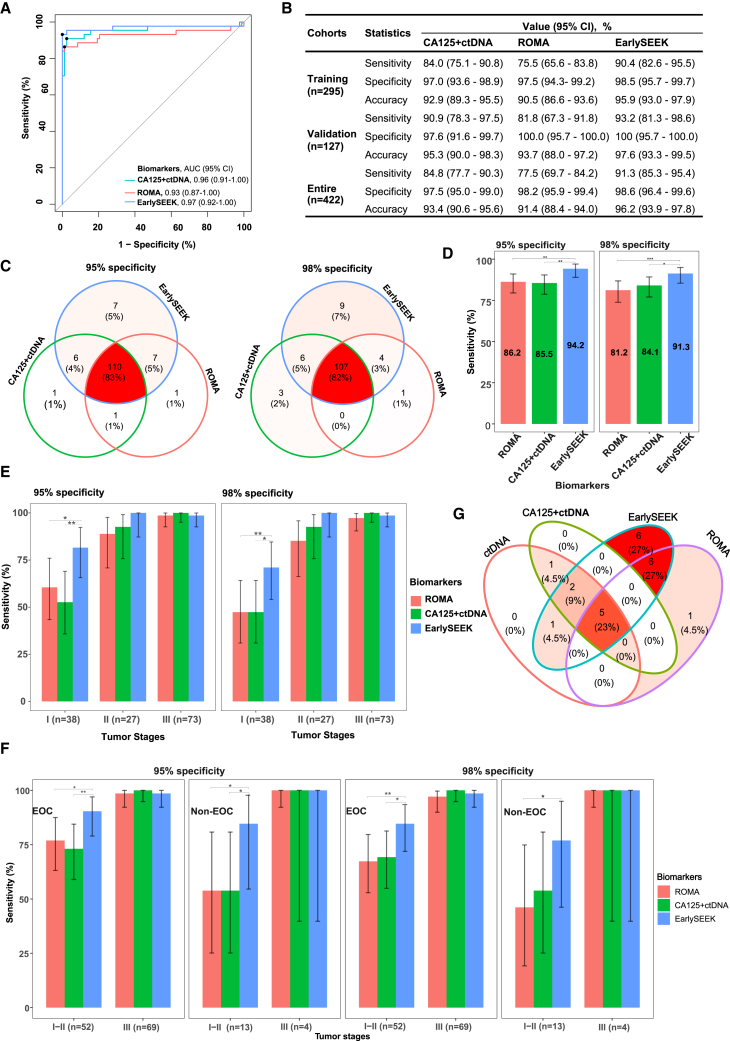
Performance comparison among the three combination tests (A) Receiver operator characteristic (ROC) curves for the ROMA, CA125+ctDNA, and EarlySEEK tests determined using the validation dataset. (B) Comparison of sensitivity, specificity, and accuracy between the ROMA model and the two combined biomarkers in different datasets. The cutoff values for the ROMA model in the premenopausal and postmenopausal populations were 11.4 and 29.9, respectively. The Youden Index was used to determine the threshold for the CA125+ctDNA and EarlySEEK and models. (C) Venn diagram showing the number and proportion of patients with OC identified based on the ROMA, CA125+ctDNA, and EarlySEEK tests at 95% and 98% specificity. (D) Overall sensitivities of the ROMA, CA125+ctDNA, and EarlySEEK combination tests at 95% and 98% specificity in the entire cohort. (E) Sensitivities of the ROMA, CA125+ctDNA, and EarlySEEK models stratified by tumor stage at 95% and 98% specificity. (F) Sensitivities of the ROMA, CA125+ctDNA, and EarlySEEK models at 95% and 98% specificity in different tumor types and at different tumor stages. (G) Identification of different indicators in patients with OC who tested negative for CA125 (<35 U/mL) at 95% specificity. The black lines indicate the 95% confidence intervals for sensitivity. ∗*p* < 0.05; ∗∗*p* < 0.01; ∗∗∗*p* < 0.001. Groups with statistical differences are labeled.

The risk of ovarian malignancy algorithm (ROMA) index, which combines CA125 and HE4 levels is also commonly used in clinical practice. Considering EarlySEEK and ROMA all included the indicators of CA125 and HE4, we used it as a control model in this study. In the validation set, the AUC value for the EarlySEEK model reached 0.97 (95% CI: 0.92–1.00) (Figure 4A), with a sensitivity of 93.2% (95% CI: 81.3%–98.6%) at 100% specificity (95% CI: 95.7%–100%) and 97.6% (95% CI: 93.3%–99.5%) accuracy (Figure 4B). The EarlySEEK model showed a higher sensitivity (McNemar’s test, *p =* 0.03), NPV (McNemar’s test, *p =* 0.03), and accuracy (McNemar’s test, *p =* 2.21E-05) than the ROMA model in the validation set at the same specificity and PPV. The accuracy of the CA125+ctDNA model was higher than that of the ROMA model (McNemar’s test, *p =* 2.75E-04) but lower than that of the EarlySEEK model in the validation set (McNemar’s test, *p =* 1.62E-03) (Table S10). In order to investigate the efficacy of EarlySEEK in the early detection of OC, we stratified OC patients in the validation set into stage I + II and stage III subgroups. We found that there were no evident differences among the three combined models in terms of AUC value, sensitivity, specificity and accuracy in stage III subgroup. However, EarlySEEK can increase the AUC value, specificity and accuracy of CA125+ctDNA model in stage I + II subgroup (Figure S4).

Because of the high specificity of the CA125+ctDNA and EarlySEEK tests, we set the test specificity to 95% and 98%, respectively, and found that the EarlySEEK model could identify 14 and 13 cases missed by the CA125+ctDNA test at the specified specificity, respectively; the EarlySEEK model could identify 13 and 15 cases missed by the ROMA test at the specified specificities, respectively; in addition, there was an overlap of approximately 80% of detected cases in the three combination tests, with 5 and 8 patients not being detected by any of the tests at the specified specificity, respectively (Figure 4C). At 98% specificity, the overall sensitivities of the ROMA, CA125+ctDNA, and EarlySEEK models were 81.2% (95% CI: 73.8%–86.8%), 84.1% (95% CI: 77.0%–89.2%), and 91.3% (95% CI: 85.4%–95.0%), respectively; the EarlySEEK model exhibited a better sensitivity than ROMA (McNemar’s test, *p =* 5.0E-04) and CA125+ctDNA (McNemar’s test, *p =* 0.012) models, while the sensitivity of the ROMA model was similar to that of the CA125+ctDNA model (McNemar’s test, *p =* 0.29); the same trend was observed when specificity was adjusted to 95% (Figure 4D). As opposed to the CA125+ctDNA model, the detection rate of the EarlySEEK model in patients with stage I OC significantly improved from 52.6% to 81.6% (McNemar’s test, *p =* 0.002) at 95% specificity and from 47.4% to 71.1% (McNemar’s test, *p =* 0.012) at 98% specificity; the sensitivity of the EarlySEEK model was also higher than that of the ROMA model in stage I patients (McNemar’s test, *p =* 0.011 at 95% specificity, *p =* 0.007 at 98% specificity) (Figure 4E). In stage II and III patients with OC, the sensitivities of the three combination tests did not significantly differ at both specificities (Figure 4E). The EarlySEEK model showed improved detection sensitivity for early-stage OC in both the EOC and non-EOC groups (Figure 4F). Furthermore, of the 27 patients with OC negative for CA125 (CA125 in the normal range: <35 U/mL), nine (33.3%) were identified by ctDNA alone, eight (29.6%) by CA125+ctDNA, twelve (44.4%) by ROMA, and twenty (74.1%) by EarlySEEK; five cases were not detected by any of the four models (specificity was adjusted to 95%, Figures 4G; Table S11).

### The performance of combination tests was not affected by menopausal status

OC is more common in postmenopausal women than in premenopausal women. CA125 performed better for OC detection in the postmenopausal population than in the premenopausal population.^39^ In this study, the performance of CA125 for OC detection in women aged ≥50 years was superior to that in women aged <50 years, with a significantly enhanced specificity (Chi-squared test, *p =* 0.003), PPV (Chi-squared test, *p =* 3.0E-04), and accuracy (Chi-squared test, *p =* 0.01) (Table 2). The ROMA and EarlySEEK tests both exhibited better sensitivity in individuals aged above 50 years than in those younger than 50 years; however, this difference was not statistically significant (Table 2). The CA125+ctDNA, ROMA, and EarlySEEK models showed similar specificity and PPV in the premenopausal and postmenopausal populations (Table 2). The EarlySEEK and CA125+ctDNA models outperformed CA125, with significant differences in specificity, PPV, and accuracy in both the premenopausal and postmenopausal groups. However, the EarlySEEK model exhibited a significantly higher sensitivity and NPV than the ROMA model in the two age groups (Table 2).

**Table 2:** Performance comparison of different biomarkers between premenopausal and postmenopausal groups

Indicators	Statistics	Premenopausal (age <50) (*n* = 130)				Menopause (age ≥50) (*n* = 292)				*p* values
		Value (95% CI)	Versus CA125+ ctDNA	Versus ROMA	Versus EarlySEEK	Value (95% CI)	Versus CA125+ctDNA	Versus ROMA	Versus EarlySEEK	
CA125	Sensitivity	74.3 (56.7–87.5)	0.083	0.083	0.180	82.5 (73.8–89.3)	0.257	0.706	0.001	0.29
	Specificity	84.2 (75.3–90.9)	0.001	0.002	0.0003	94.7 (90.5–97.4)	0.01	0.008	0.005	0.003
	PPV	63.4 (51.2–74.2)	0.001	0.003	0.0003	89.5 (82.2–94.0)	0.013	0.009	0.004	0.0003
	NPV	89.9 (83.4–94.0)	0.03	0.41	0.08	90.9 (86.7–93.8)	0.19	0.91	0.001	0.79
	Accuracy	81.5 (73.8–87.8)	0.0003	0.025	0.0002	90.4 (86.4–93.5)	0.012	0.11	0.00002	0.01
CA125+ ctDNA	Sensitivity	82.9 (66.4–93.4)	–	0.014	1.000	85.4 (77.1–91.6)	–	0.157	0.007	0.71
	Specificity	96.8 (91.1–99.3)	–	0.65	0.56	97.9 (94.7–99.4)	–	0.56	0.32	0.69
	PPV	90.6 (75.9–96.8)	–	0.85	0.57	95.7 (89.3–98.3)	–	0.63	0.26	0.37
	NPV	93.9 (88.1–97.0)	–	0.016	0.98	92.5 (88.5–95.2)	–	0.17	0.006	0.66
	Accuracy	93.1 (87.3–96.8)	–	0.13	0.74	93.5 (90.0–96.0)	–	0.37	0.005	0.87
ROMA	Sensitivity	65.7 (47.8–80.9)	0.014	–	0.034	81.6 (72.7–88.5)	0.157	–	0.0003	0.052
	Specificity	97.9 (92.6–99.7)	0.65	–	1.00	98.4 (95.4–99.7)	0.56	–	0.32	1.00
	PPV	92.0 (74.1–97.9)	0.85	–	0.82	96.6 (90.1–98.9)	0.63	–	0.21	0.31
	NPV	88.6 (83.0–92.5)	0.016	–	0.03	90.7 (86.7–93.6)	0.17	–	0.0003	0.55
	Accuracy	89.2 (82.6–94.0)	0.13	–	0.08	92.5 (88.8–95.2)	0.37	–	0.0002	0.27
EarlySEEK	Sensitivity	82.9 (66.4–93.4)	1.000	0.034	–	94.2 (87.8–97.8)	0.007	0.000	–	0.07
	Specificity	97.9 (92.6–99.7)	0.56	1.00	–	98.9 (96.2–99.9)	0.32	0.32	–	0.60
	PPV	93.6 (78.5–98.3)	0.57	0.82	–	98.0 (92.4–99.5)	0.26	0.21	–	0.24
	NPV	93.9 (88.2–97.0)	0.98	0.03	–	96.9 (93.5–98.6)	0.006	0.0003	–	0.66
	Accuracy	93.9 (88.2–97.3)	0.74	0.08	–	97.3 (94.7–98.8)	0.01	0.0002	–	0.10

CI, confidence interval; PPV, positive predictive value; NPV, negative predictive value.

Considering that sensitivity is of great importance in clinical practice, we also compared the performance of different models adjusted by sensitivity. Based on the reported sensitivities of CA125 and the ROMA model,^40^ we adjusted the sensitivity to approximately 85%, 90%, and 95% to assess the performance metrics of the different indicators. The EarlySEEK model was found to outperform all the other three models (CA125 alone, CA125+ctDNA, and ROMA) in terms of specificity and PPV, and retained a high sensitivity in both the premenopausal and postmenopausal populations (90% and 95%, respectively). To be detailed, at around 95% sensitivity, predictive models using CA125, CA125+ctDNA, and ROMA exhibited an overall specificity of 71.58%, 70.53%, and 5.26%, respectively; while the EarlySEEK model exhibited a specificity of 90.53%. The performance of the CA125+ctDNA model was not significantly different from that of CA125 alone in the premenopausal population; however, the CA125+ctDNA model exhibited a higher specificity and PPV than CA125 alone at a sensitivity of approximately 85% in the postmenopausal population (Table S12).

### Combination tests exhibit better performance in differentiating the benign and malignant tumors compared to CA125 alone

**Figure 5:**
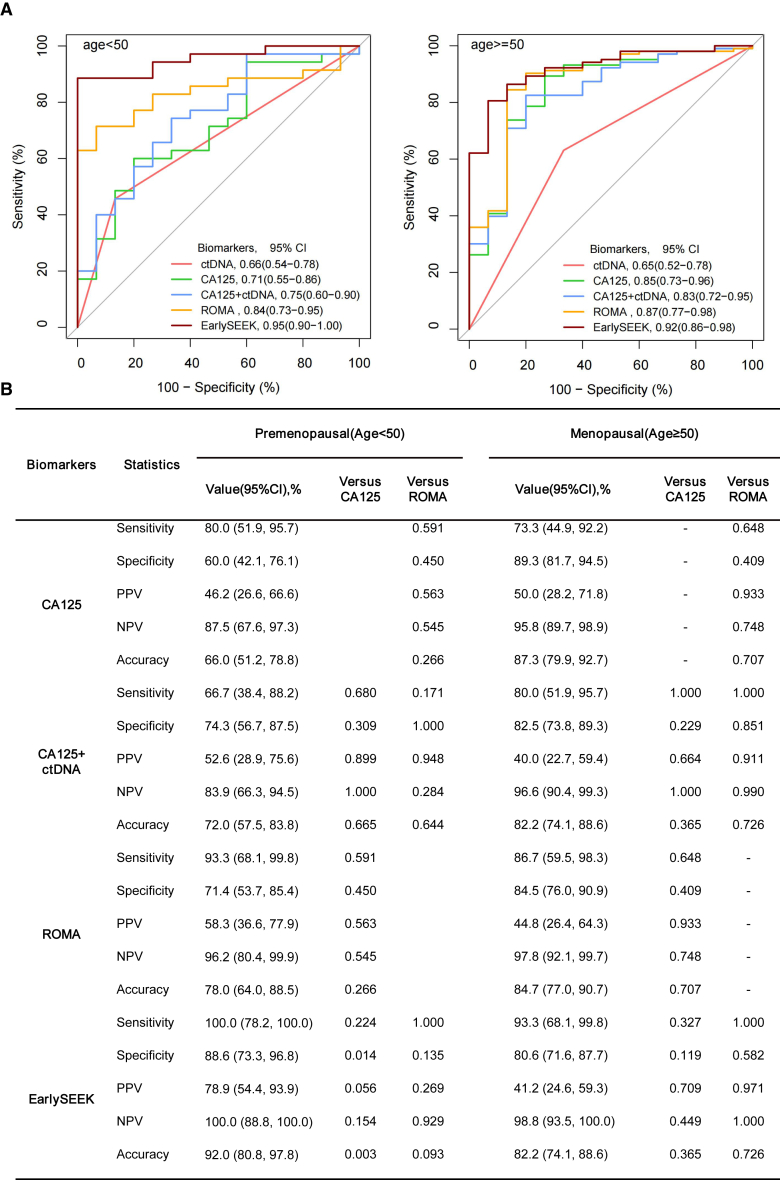
Performance comparison between different biomarkers in distinguishing benign from malignant ovarian tumors (A) ROC curves for ovarian tumors in the premenopausal (age < 50, *n* = 50) and postmenopausal (age ≥ 50, *n* = 118) populations. Different colors represent different variables or variable combinations; 15 and 35 benign and malignant ovarian tumors, respectively, were included in the premenopausal population, while 15 and 103 benign and malignant ovarian tumors, respectively, were included in the menopausal population. (B) Comparison of sensitivity, specificity, and accuracy between various biomarkers. ctDNA: circulating tumor DNA; PPV: positive predictive value; NPV: negative predictive value; ROC: receiver operator characteristics.

We assessed the diagnostic performance of the two combination tests for adnexal cysts; this analysis included 138 patients with OC and 30 patients with benign tumors. Considering that there was a significant age difference between patients with benign ovarian tumors and those with malignant ovarian tumors (t test, *p =* 0.008, Table S13), we split the group of patients into two subgroups, those aged < 50 and those aged ≥ 50 and found that there was no statistically significant difference in age between patients with benign and malignant ovarian tumors within the subgroups (Mann-Whitney U test, both *p* > 0.4, Table S13). We found the AUC values for the EarlySEEK model were higher than that for CA125 or ctDNA alone, CA125+ctDNA and ROMA both in the premenopausal and postmenopausal group (Figure 5A; Table S13). However, the statistical significance was only found in the premenopausal (*DeLong’s* test, all *p* < 0.01, Table S13), but not in the postmenopausal group (*DeLong’s* test, *p* > 0.05, Table S13). In women aged < 50 years, the EarlySEEK model exhibited comparable sensitivity, PPV, and NPV to CA125 but surpassed it in terms of specificity (Chi-squared test, *p =* 0.014) and accuracy (Chi-squared test, *p =* 0.003). Although, EarlySEEK model exhibited a higher sensitivity, specificity, PPV, NPV, and accuracy compared to the ROMA model, there was no statistical difference (Chi-squared test, all *p > 0.09*, Figure 5B). In women aged ≥50 years, the EarlySEEK model exhibited comparable specificity, PPV, NPV, and accuracy to CA125 but surpassed it in terms of sensitivity; the similar trend was observed with the CA125+ctDNA model (Figure 5B). Moreover, EarlySEEK showed similar performance with ROMA in women aged ≥50 years. To further explore the performance of our model in distinguishing early-stage OC from benign tumor, we divided the OC cohort into stage I + II group and stage III group. Because there was no statistical difference of age within the subgroup, we didn’t perform further subgroup analysis of age. In stage I + II subgroup, the EarlySEEK model also exhibited higher AUC values than other models, and it exhibited higher sensitivity and NPV and similar specificity, PPV, and accuracy to CA125 (Figures S5A and S5B). However, there was not significant difference between EarlySEEK and ROMA when considering the metrics of sensitivity and NPV (Figure S5B). These results showed that in younger patients, the EarlySEEK model could distinguish malignant tumors from benign tumors more effectively than CA125 alone or the ROMA model.

## Discussion

In this study, we report two multi-analyte predictive LR models for early OC detection integrating five protein biomarkers with cfDNA targeted deep sequencing using a panel composed of 18 frequently mutated genes in cancer patients. One model combined CA125 with ctDNA (CA125+ctDNA), while the other further integrated HE4, IL-6, PRL, and CA19-9 with CA125 and ctDNA (EarlySEEK). The EarlySEEK could overcome the weakness of low specificity and sensitivity of CA125 alone. As compared to CA125, the EarlySEEK model detected early-stage OC more effectively, potentially allowing for patients to be treated earlier, with better treatment outcomes.

The cut-off point for CA125 has a significant impact on its performance in OC screening and early detection. Selection of an appropriate cut-off point is crucial when using CA125 alone for OC detection. A low CA125 threshold increases sensitivity and decreases specificity, resulting in unnecessary surgeries and psychological consequences.^41^ A high CA125 threshold improves specificity and decreases sensitivity, which may lead to the non-detection of most early-stage cancers.^39^^,^^42^ The cut-off value for CA125 in our study on the Youden index was only 18.9, and its sensitivity and specificity for OC detection were 93.2% and 84.3%, respectively. In this cut-off point, a specificity of 84.3% may bring anxiety and unnecessary surgery to patient. So, we then set the threshold for CA125 at 35 U/mL, which is the conventional cut-off point for CA125^43^ and the upper limit for its normal detection range in detection kits. In line with the findings of previous studies, we found CA125 alone exhibited an OC detection sensitivity, specificity, and accuracy of 80.4%, 91.2%, and 87.7%, respectively.^40^^,^^44^ CA125 exhibited a sensitivity of 74.3% (at 84.2% specificity) and 82.5% (at 94.7% specificity) in patients aged <50 years and in those aged ≥50 years, respectively (Table 2).

Previous studies have shown that ctDNA testing exhibits a high specificity for OC detection; however, its sensitivity ranges from 27% to 100% because ctDNA detection rate can be influenced by multiple factors such as detection technologies, sample size, and tumor stage.^45^ Targeted sequencing and high-throughput sequencing methods (such as whole exome sequencing or whole genome sequencing) are the most commonly used methods for ctDNA detection. In our study, we utilized an 18-gene panel to detect ctDNA mutations for at least three key reasons. First, high-throughput sequencing methods can get a more comprehensive picture of all the mutations, as well as copy number variation in tumor, but it generally work in the range of 5%–10% allelic frequencies. Targeted sequencing is more sensitive than high-throughput sequencing method, with a lower limit of a few hundreds or thousands of a percent when optimized.^46^^,^^47^ Considering that the amount of ctDNA is limited, it is better to use target sequencing to measure the ctDNA mutation status. Second, target-sequencing, using 18-gene panel in our work, is more fitful for clinical research to explore the clinical application potential, while high-throughput sequencing methods are more fitful for basic research to identify new mutation variation of cancer.^48^ Third, target sequencing can be divided into two categories, capture through hybridization and PCR amplification. In our work, we used an amplicon-based NGS method, CLAmp-seq, for ultrasensitive detection of low mutant allele frequency (MAF) variants which achieves high conversion rate and low background noise with a simple amplicon workflow.^49^^,^^50^

Considering that the CancerSEEK study,^31^ the PapSEEK study,^27^ the study by Maritschnegg et al.,^26^ and our study all used small panels with less than 20 genes to reflect ctDNA detection rate(Table S14), we use these three studies as a control. As for the ctDNA detection rate, we detected ctDNA mutations at a rate of 58.7% as compared to 43% in the PapSEEK study,^27^ 80% in the study Maritschnegg et al.^26^, and 100% in the CancerSEEK study.^31^ The pathogenic ctDNA variants detected in our work were consistent with previously reported variants in the catalog of somatic mutations in cancer (COSMIC: https://cancer.sanger.ac.uk/cosmic/) database. Given that *TP53* is the gene with the highest frequency of mutations in individuals with OC, we selected *TP53* as a representative example and identified that the majority of variants detected were hot variants commonly associated with cancer, such as R248 and R282. The *TP53* mutation rate was 42% in our study as compared to 33.7% in the PapSEEK study,^27^ 46.7% in the study by Maritschnegg et al.,^26^ and 85.2% in the CancerSEEK study.^31^ In the study, we found that the ctDNA detection rate is influenced by tumor stage and tumor histological characteristics. First, we found the ctDNA detection rate was higher in stage III OC compared to that in early-stage one (Figure 2D) which may result from the higher tumor burden and extent of metastatic spread in stage III OC. Second, we found the ctDNA detection rate in the SC histological subtype to be significantly higher than that in the CCC and EC histological subtypes, but with no statistically significant difference found between other histological subtypes. Our EOC cohort included 76.9% (93/121) patients with SC, 12.4% (15/121) patients with CCC, 6.6% (8/121) patients with EC, and 4.1% (5/121) patients with MC; these proportions are similar to those found in previous studies.^51^ The difference of ctDNA detection rate among various subtypes may due to the difference in the proportion of early-stage OC, tumor size, and concordance rate between ctDNA mutations and matched tumor samples. To be detailed, the ctDNA detection rate for the MC histological subtype was 80%, and this was highest among all the OC subtypes (Figure 2E). This may be due to the larger tumor size of MCs^52^ as ctDNA detection rates are higher in patients with larger tumor volumes.^51^ Meanwhile, the higher concordance rate between ctDNA mutations and matched FFPE tumor samples in HGSC compared to other subtypes^32^ may contribute to the high ctDNA detection rate in HGSC which was consistent with other studies.^45^ Furthermore, in this study, 35.5% (33/93), 60% (9/15) and 62.5% (5/8) of patients with SC, CCC, and EC, respectively, were in the early stage which may lead to the low ctDNA detection rate in EC and CCC. However, given the small number of patients with EC and MC, more samples are needed to clarify whether there exists an actual difference in ctDNA mutation detection rate between the two subtypes.

The origin of ctDNA mutation is of great significance in the study related to ctDNA. We performed whole exon sequencing on paired tumor biopsies in 32 OC patients. The overall concordance rate between plasma ctDNA and tumor tissues is 68.8%, which was consistent with other studies.^27^ As for *TP53* gene, the *TP53* mutation concordance rate between plasma and tumor biopsies is 68% (17/25), which was comparable to other studies ranges from 61% to 100%.^53^^,^^54^To be detailed, 17 patients can detected *TP53* mutation both in plasma and tissue, six patients who had *TP53* mutations detectable in paired tissue did not have the identical mutations in plasma, two patients who had *TP53* mutations detectable in plasma did not have the identical mutations in their primary tumors. Four reasons may lead to the discordance between ctDNA and paired tumor biopsies. First, the technical artifacts and limitation. Considering that the mutations identified in plasma were hot variants in cancer and the high specificity of ctDNA in our assays (95.2% in the validation cohort), we think technical artifacts may not be the main reason contributed to the negative detection of mutation in the tumor biopsies compared to plasma. However, considering we only sequenced target sequences in plasma but performed whole exon sequencing in tumor biopsies, technical difference may lead to the negative detection of mutation in the plasma compared to paired tissue. Second, the tumor heterogeneity within the tumor and the heterogeneity between primary and metastatic tumor. It is reported that the concordance rate was higher in metastatic biopsies compared to primary ones.^54^ OC is of high tumor heterogeneity but we only sequenced a small portion of the primary tumors, which may lead to this discordance. Third, sub-clonal mutation which may be the most important. In our work, we compared predicted probability and performance between data with clonal hematopoiesis of indeterminate potential (CHIP) variants (ctDNA) filtered and data with CHIP variants (cfDNA) retained and found that the omission of CHIP filtering may lead to an increase in the predicted probability of cancer detection, particularly in healthy individuals; however, this did not affect our final evaluation. Fourth, other reasons such as a low tumor load in early-stage OC can also lead to the discordance between plasma and paired tumor biopsies.

Although ctDNA analysis has been considered as a promising approach for OC detection, using ctDNA alone is not sufficient for the early OC detection.^45^^,^^55^ Detecting cancer patients at early stage with ctDNA mutation alone might be challenging, especially when the tumor diameter is less than 10 mm, as ctDNA levels may be too low to be detected.^56^ In our work, ctDNA alone can only exhibit a sensitivity of 61.4% at 95% specificity in the validation cohort (Figure 3). Additional research is required to confirm the value of use ctDNA alone for OC detection. We combined CA125 with ctDNA to make up the shortage of low sensitivity of ctDNA alone and found although CA125+ctDNA exhibited enhanced PPV, NPV, and accuracy compared to CA125 and ctDNA detection alone in the entire cohort (Table S6); its sensitivity for stage I OC detection was found to be inadequate (Figure 3E). Moore et al. established the multivariate index, ROMA, using an LR model that included CA125 and HE4 serum levels, as well as menopausal status.^57^ The ROMA model was found to predict OC better than CA125 or HE4 alone^58^; however, menopausal status alters its sensitivity and specificity as this model exhibits a higher sensitivity in postmenopausal women than in premenopausal women.^59^ In other studies, the integration of additional protein biomarkers with CA125 or HE4 was investigated for early OC detection. A multivariate model including five proteins (CA125, osteopontin, HE4, leptin, and PRL) was found to have an AUC value of 0.996, outperforming CA125 alone; however, only 25 patients with benign tumors and 18 patients with OC were analyzed in this study.^22^ Han et al. evaluated the efficacy of 12 serum biomarkers for detecting EOC and found that the combination of CA125, HE4, E-cadherin, and IL-6 had the highest AUC value i.e., 0.96; however, 84.8% of patients with OC were found to be in the advanced stage.^23^ CA125 in combination with CA19-9 and six other biomarkers (epidermal growth factor receptor, granulocyte colony stimulating factor, eotaxin, IL-2 receptor, vascular cell adhesion protein, and migration inhibitory factor) exhibited a sensitivity of 98.2% at a specificity of 98.7% for early-stage OC detection,^57^ however, some of these biomarkers are not routinely analyzed in clinical settings. Based on these findings, we incorporated more protein biomarkers to CA125 and ctDNA and found that the EarlySEEK model, which integrates ctDNA detection with the five proteins, CA125, HE4, PRL, IL-6, and CA19-9, demonstrated the highest accuracy and F1 score (Table S9), with a sensitivity of 71.05% and 100% at 98% specificity in patients with stage I and II OC, respectively (Figure 4E). Other cancer biomarkers, such as metabolomes,^17^ RNAs,^60^^,^^61^ and methylomes,^62^ could be combined in a similar way for early OC detection. A recently published study tested the metabolomic features of uterine fluid for early OC detection, and found an AUC value similar to that obtained for the EarlySEEK model (0.957 versus 0.97). However, this study did not include healthy women in the control group. In addition, the study only compared AUC between CA125 and the ROMA model, but did not report on the sensitivity, specificity, accuracy, PPV, and NPV of the assay.^17^ An effective screening test for early-stage OC detection is considered to have a PPV of at least 10% when its sensitivity is above 75% and its specificity is at least 99.6%.^63^ In our validation dataset, using the Youden index, the EarlySEEK model was found to exhibit a sensitivity of 93.2% and a specificity of 100% (Figure 4B), implying that it could potentially be used for OC screening to improve patient survival.

As concerns the two models we developed in this study, the overall performance of the EarlySEEK model was better than that of the CA125+ctDNA model. When overall specificity was adjusted to 95% (exactly 95.1%), the sensitivity of the CA125+ctDNA model was 85.5%, while that of the EarlySEEK model was 94.2% (*p =* 0.003) (Figure 4D). When sensitivity was set to 95%, the specificity of the CA125+ctDNA and EarlySEEK models was 70.5% and 90.5% (*p =* 0.0001), respectively, in the premenopausal population, and 80.4% and 97.9% (*p =* 9.2E-09), respectively, in the postmenopausal population (Table S12). When the Youden index was used to set the optimal cut-off point, the two models exhibited similar performance metrics in the premenopausal population; however, in the postmenopausal population, the sensitivity, NPV, and accuracy of the EarlySEEK model were higher than those of the CA125+ctDNA model (Table 2). Moreover, in patients with a negative CA125 test (<35 U/mL), the OC detection rates for the CA125+ctDNA and EarlySEEK models were 29.6% and 74.1% (Figure 4G), respectively, at 95% specificity; and 25.9% and 63%, respectively, at 98% specificity (Table S11). The turnaround time for the two tests was almost the same. The cost of running the CA125+ctDNA test is approximately $60 less than that for running the EarlySEEK test. As concerns the clinical application of the two models, to select a model, there is need to comprehensively consider patient age or menopausal status, CA125 level, and patient economic status, as well as the availability of tests for the different indicators in local hospitals and other medical results. For example, (1) considering that EarlySEEK outperformed CA125 or ctDNA alone, CA125+ctDNA and ROMA in many ways and the turnover time for these tests was similar, EarlySEEK is the first choice when the patients’ economic situation permits. (2) Considering that CA125+ctDNA can largely elevate the specificity, PPV, NPV, and accuracy of CA125 alone, and the accuracy of CA125+ctDNA can reach to 93.1% in premenopausal women, CA125+ctDNA is a better choice, especially for those in hospitals unable to detect HE4, PRL, or IL-6. (3) Considering that the diagnostic performance among CA125 or ctDNA alone, CA125+ctDNA, and ROMA was not significantly different and CA125 alone exhibited high specificity and accuracy in postmenopausal women, CA125 alone may be sufficient for patients with significantly elevated levels. However, if patients’ economic situation permit, EarlySEEK can be a better choice for those persons. (4) In premenopausal women, especially women of childbearing age, if an adnexal mass is present and the patient is unwilling to undergo biopsy or surgery, EarlySEEK can be considered for follow-up. (5) Considering the similar performance of ROMA and EarlySEEK in differentiating benign and malignant OC in postmenopausal, for older patients with adnexal-area masses, ROMA is preferred.

In conclusion, EarlySEEK is a non-invasive method for improving early-stage OC detection. The EarlySEEK model had a sensitivity of 93.2% and a specificity of 100% and its performance in detecting early-stage OC was unaffected by age or menopausal status. Moreover, this model can also be used to differentiating the benign and malignant adnexal cysts, with an overall accuracy of over 90% in menopausal women. Thus, the EarlySEEK model can be used to detect early-stage OC, thereby increasing patient survival.

### Limitations of the study

First, it was observed that tumor stages and histological characteristics may influence the ctDNA detection rate, subsequently affecting the performance of the models. The limited number of patients in the study, particularly those in the early stages, restricted the ability to perform more comprehensive subgroup analyses. Furthermore, while categorizing both stage I and stage II OC patients as early-stage OV aligns with clinical guidelines, literature, and routine practice, significant biological differences exist between these stages, which may impact the molecular detection methods employed. Additionally, although this study included a relatively larger sample size of patients with benign tumors compared to similar studies to better evaluate model performance, the overall sample size was still limited. A larger cohort of patients with benign tumors is necessary to further validate the models’ ability to distinguish between benign and malignant tumors.

Second, we employed targeted sequencing rather than full-length sequencing to detect ctDNA mutations, which may have resulted in the omission of important information, such as copy number variations. Given that copy number variations play a crucial role in the early detection of cancer, future studies utilizing full-length sequencing would enhance the credibility of these findings.

Third, this was a retrospective study, and the developed models need to be validated using larger prospective, multi-center datasets. Additionally, the performance of the EarlySEEK model requires further validation in an external cohort. While we used CancerSEEK data to validate the CA125+ctDNA model, it is important to note that the ctDNA detection method employed in the CancerSEEK study differed from the one used in our study. Despite this, the performance of both models was comparable (Figures 3B and 3H). However, due to differences in the units of measurement for HE4 and PRL between the CancerSEEK study and our study, we were unable to use their data for external validation of the EarlySEEK model. If we had developed a logistic regression (LR) model using the same set of variables as the CancerSEEK study (CA125+ctDNA+HE4+PRL+IL-6+CA19-9), we would have achieved a sensitivity of 98.15%, a specificity of 98.93%, and an accuracy of 98.81% (Table S6), which are consistent with the results obtained in our study.

## Resource availability

### Lead contact

Further information and requests for resources and reagents should be directed to and will be fulfilled by the lead contact, Xiaodong Cheng (chengxd@zju.edu.cn).

### Materials availability

This study did not generate any new unique reagents.

### Data and code availability

All data associated with this study have been deposited in the Genome Sequence Archive (Genomics, Proteomics & Bioinformatics 2021) in National Genomics Data Center (Nucleic Acids Res 2022), China National Center for Bioinformation/Beijing Institute of Genomics, Chinese Academy of Sciences (GSA-Human: HRA007484 and HRA007491) that are publicly accessible at https://ngdc.cncb.ac.cn/gsa-human.

The original code generated in this study has been deposited at Science DataBank and is publicly available as of the date of publication. The DOI is listed in the key resources table.

Any additional information required to reanalyze the data reported in this paper is available from the lead contact upon request.

## Acknowledgments

We thank Yun Liang and Xiaofei Zhang for their assistance in pathological diagnosis (Women’s Hospital, Zhejiang University School of Medicine, China), and Qianqian Yao (Shanghai YunSheng Medical Laboratory Co., Ltd.) for assisting with the technical aspects of the study and data analysis. We also extend our thanks to the Biobank of the Women's Hospital, School of Medicine at Zhejiang University for their support in collecting samples. This work was supported by the 10.13039/501100017599Science and Technology Program of Zhejiang Province, the 10.13039/501100008990Department of Science and Technology of Zhejiang Province (grant no. 2024C03159), the 10.13039/501100012166National Key R&D Program of China (grant no. 2022YFC2704200 and 2022YFC2704203), the 10.13039/501100001809National Natural Science Foundation of China (grant no. 82273348 and 82072858), and the 10.13039/501100012226Fundamental Research Fund for the Central Universities (grant no. 2021FZZX001-44). We would like to thank Editage (www.editage.cn) for English language editing.

## Author contributions

F.W., L.W., Z.X., and X. Chen conducted the experiments and wrote the paper; L.L., T.Z., Z.S., L.Z., J.D., F.Q., and S.Y. collected the samples and patient information; Y.L., Z. L., and S.Q. carried out data analysis; X. Cheng designed the experiments; All the authors discussed the results, reviewed the draft manuscript, and approved the final version of the manuscript.

## Declaration of interests

The authors declare no competing interests.

## STAR★Methods

### Key resources table

**Table:** 

REAGENT or RESOURCE	SOURCE	IDENTIFIER
**Biological samples**		

Plasma, WBCs and tissue	Women’s Hospital, School of Medicine, Zhejiang University	https://zju.womanhospital.cn/

**Chemicals, peptides, and recombinant proteins**		

*CircLigase II ssDNA ligase*	*EpiCentre*	*Cat#CL9021K*
exonuclease	*NEB*	*Cat#M0293s*
Phi29 DNA polymerase	NEB	*Cat#M0269S*

**Critical commercial assays**		

LBgard Blood Tubes	Biomatrica	*Cat#*68021-001
MagicPure® Cell-Free DNA Kit II	Transgen Biotech	Cat#EC211-01
Agilent High Sensitivity DNA Kit	Agilent	*Cat#5067-4626*
Qubit dsDNA HS Assay Kit	Life Technologies	*Cat#Q32854*
QIAamp DNA Blood Mini Kit	QIAGEN	*Cat#*51104
QIAamp DNA FFPE Tissue Kit	QIAGEN	*Cat#*56404
AMPure beads	Beckman Coulter	*Cat#A63881*
MGISEQ-2000RS high-throughput sequencing reagent set	MGI	*Cat#1000013853*
NEBNext Ultra II End Repair/dA-Tailing Module	NEB	*Cat#E7546L*
NEBNext Ultra II Ligation Module	NEB	*Cat#*E7595L
KAPA Hyper Prep Kit	KAPA Biosystems	*Cat#KK8504*
MGISEQ-2000RS High-Throughput Sequencing Reagent Kit (PE150	MGI	*Cat#1000012555*
CA125/HE4/CEA/CA19-9/IL-6/PRL/AFP	Roche Diagnostics	Cobas e411

**Deposited data**		

Ovarian cancer mutation data	TCGA	https://www.cancer.gov/ccg/research/genome-sequencing/tcga
Ovarian cancer mutation data	COSMIC	https://cancer.sanger.ac.uk/cosmic
Human reference genome NCBI build 37, GRCh37	Genome Reference Consortium	http://www.ncbi.nlm.nih.gov/projects/genome/assembly/grc/human/
The results of Whole exon sequencing in tumor biopsies	China National Center for Bioinformation/Beijing Institute of Genomics, Chinese Academy of Sciences	https://ngdc.cncb.ac.cn/search/specific?db=hra&q=HRA007484
ctDNA results in plasma	China National Center for Bioinformation/Beijing Institute of Genomics, Chinese Academy of Sciences	https://ngdc.cncb.ac.cn/search/specific?db=hra&q=HRA007491
Codes	Science Data Bank	https://doi.org/10.57760/sciencedb.09338

**Software and algorithms**		

Burrows-Wheeler Aligner	/	https://bio-bwa.sourceforge.net/
FreeBayes	Github	https://github.com/freebayes/freebayes
SAMBLASTER	Github	https://github.com/GregoryFaust/samblaster
Picard tools	Broad Institute	http://broadinstitute.github.io/picard/
Mutect2	Broad Institute	https://gatk.broadinstitute.org/hc/en-us/articles/360037593851-Mutect2
ANNOVAR	ANNOVAR	https://annovar.openbioinformatics.org/en/latest/
The R Foundation for Statistical Computing	The Comprehensive R Archive Network	version 4.2.2, https://www.r-project.org/
epiDisplay package	The Comprehensive R Archive Network	https://cran.r-project.org/web/packages/epiDisplay/epiDisplay.pdf
ggVennDiagram package	The Comprehensive R Archive Network	https://cran.r-project.org/web/packages/ggVennDiagram/readme/README.html
Openxlsx package	The Comprehensive R Archive Network	https://cran.r-project.org/web/packages/openxlsx/index.html
pROC package	The Comprehensive R Archive Network	https://cran.r-project.org/web/packages/pROC/pROC.pdf
ggplot2 package	The Comprehensive R Archive Network	https://cran.r-project.org/web/packages/ggplot2/index.html
gmodels package	The Comprehensive R Archive Network	https://cran.r-project.org/web/packages/gmodels/index.html
DTComPair package	The Comprehensive R Archive Network	https://cran.r-project.org/web/packages/DTComPair/index.html
cowplot package	The Comprehensive R Archive Network	https://cran.r-project.org/web/packages/cowplot/index.html
Python	Python Software Foundation	version 3.9, https://www.python.org/downloads/release/python-390/
sklearn	Python Software Foundation	version 1.3, https://scikit-learn.org/stable/
Logistic Regression	Python Software Foundation	https://scikit-learn.org/stable/modules/generated/sklearn.linear_model.LogisticRegression.html#sklearn.linear_model.LogisticRegression
Diagnostic test evaluation calculator	MedCalc	https://www.medcalc.org/calc/diagnostic_test.php

### Experimental model and study participant details

#### Human specimens

The blood samples were obtained from 138 patients with OC, 314 controls consisting of 30 patients with benign tumors and 284 healthy individuals (female, aged from 27 to 82 years old) at Department of Gynecologic Oncology, Women’s Hospital, School of Medicine, Zhejiang University from December 2018 to August 2021. The cancer tissue samples were obtained from 32 patients with OC (female, aged from 37 to 77 years old) at Department of Gynecologic Oncology, Women’s Hospital, School of Medicine, Zhejiang University from December 2018 to August 2021.The study was approved by the Institutional Review Board of the Women’s Hospital School of Medicine of Zhejiang University (ID: 20180196).Inclusion and exclusion criteria are listed in Table S15. Histological type and disease stage were determined based on the 2018 guidelines of the International Federation of Gynecology and Obstetrics (FIGO) for all malignant cases.

### Method details

#### Sample collection

LBgard blood tubes (Biomatrica, San Diego, USA) were used to collect 6 mL of blood from both patients with OC or individuals suspected to have OC and healthy controls (n = 500). For patients of OC or those suspected to have OC, blood samples were collected before anesthesia. Within 3 days after drawing blood samples, the samples were centrifuged at 1,900 × *g* for 10 min at 4°C to separate WBCs from the middle phase. To remove the remaining debris, plasma was collected from the top phase and re-centrifuged at 16,000 × *g* for 10 min at 4°C. Primary tumor tissues were collected from patients with OC. Plasma and WBCs were stored at –80°C until use. Three participants were excluded due to hemolysis of their blood samples (Figure S6).

#### DNA extraction

cfDNA was isolated from 2–4 mL of plasma (n = 497) using the MagicPure® Cell-Free DNA Kit II (Transgen Biotech, Beijing, China) and rinsed in 80 μL of Tris-EDTA buffer, according to the instructions of the manufacturer. The quality of the extracted cfDNA was evaluated using a Qubit 3.0 Fluorometer (Life Technologies, Grand Island, NY, USA) with a Qubit dsDNA HS Assay Kit (Life Technologies) and a 2100 Bioanalyzer (Agilent Technologies, Palo Alto, CA, USA) with an Agilent High Sensitivity DNA Kit. The QIAamp DNA Blood Mini Kit (QIAGEN, Hilden, Germany) was used to extract genomic DNA (gDNA) from WBCs (n = 92). The QIAamp DNA FFPE Tissue Kit (QIAGEN) was used to collect gDNA from tumor tissues. Four participants were excluded due to insufficient cfDNA (< 5 ng) or severe cfDNA contamination by gDNA (Figure S6). All DNA samples were stored at –80°C until use.

#### Design of the detection panel

Genes frequently mutated in cancer patients were searched and identified using The Cancer Genome Atlas (TCGA) and Catalogue of Somatic Mutations in Cancer (COSMIC) databases for the design of targeted deep sequencing panels (Table S16). A collection of protein biomarkers, including CA125, HE-4, CA19-9, CEA, PRL, HE4, IL-6, and AFP, was put in place through a literature review.^22^^,^^23^^,^^31^^,^^34^^,^^35^^,^^36^^,^^37^^,^^38^ CA125, HE4, CEA, CA19-9, and AFP are commonly used tumor biomarkers in clinical practice. IL-6 and PRL expression is also associated with ovarian tumors.

#### cfDNA sequencing

Plasma cfDNA (n = 452) was sequenced using CLAmp-SEQ (circular ligation and amplification sequencing)^50^ (Yunsheng Medical Laboratory, Shanghai, China), a amplicon-based Next-generation sequencing (NGS) workflow tool, using an 18-gene panel.^33^ Briefly, double-stranded DNA (dsDNA) molecules were denatured to form single-stranded DNA (ssDNA) molecules with an input of 10–30 ng cfDNA in a 20-μL reaction medium; then the ssDNA was circularized by intramolecular ligation using CircLigase II ssDNA ligase (*EpiCentre*) and the NEBNext Ultra II Ligation Module (NEB). Uncircularized ssDNA was eliminated using exonuclease (NEB). Next, circularized DNA was amplified through direct rolling circle amplification (RCA) using Phi29 DNA polymerase (NEB) and primers for target genes as part of sequencing adaptors. The second round of PCR was performed to amplify RCA molecules using full-length sequencing adaptors consisting of sample indices. The products of the first and second rounds of PCR were purified using AMPure beads (Beckman Coulter). cfDNA libraries were sequenced on a MGISEQ-2000 device (MGI Tech, Shenzhen, China) using the MGISEQ-2000RS high-throughput sequencing reagent set (SE400, MGI) to generate 400-bp single-end reads (n = 434), or using the MGISEQ-2000RS high-throughput sequencing reagent set (FCL PE200, MGI) to generate 200-bp paired-end reads (n = 10) or on Illumina HiSeq 2500 device to generate 250-bp paired-end reads (n = 8). Two participants were excluded due to sequencing data quality control failure.

#### WBC gDNA sequencing

WBC gDNA (n = 92) was sequenced using the same panel for participants positive for cfDNA mutations (n = 46) and for those negative for cfDNA mutations as controls (n = 46). WBC sequencing was performed to filter out somatic mutations from CHIP. These somatic mutations are most frequently observed in samples of older patients^64^ with a higher risk of developing cancer. Then, gDNA was sonicated into short fragments, with a peak at approximately 200 bp, using an M220 ultrasonicator (Covaris, Woburn, MA, USA), and 100 ng of the fragmented gDNA was used for library construction using the KAPA Hyper Prep kit (KAPA Biosystems, Boston, MA, USA) according to the instructions of the manufacturer. In brief, dsDNA was subjected to end repair and A-tailing processes using the NEBNext Ultra II End Repair/dA-Tailing Module (NEB) followed by 1.8X bead purification. Then, an adapter was introduced to the ligated product, which was bead-purified again. Next PCR amplification was carried out using the Illumina primers, P5 and P7. Prior to panel capture, amplified genomic DNA was cleaned using AMPure beads. The gDNA libraries were sequenced on a MGISEQ-2000 device (MGI Tech) in the 400 bp single-end mode (n = 80) or paired-end 200 bp mode (n = 4) or paired-end 250 bp mode on Illumina HiSeq 2500 (n = 8).

#### Sequencing data processing

Variant calling was performed as previously described.^33^^,^^50^ Sequencing reads were aligned against the human reference genome (hg19/GRCh37) using the Burrows-Wheeler Aligner platform (http://bio-bwa.sourceforge.net/bwa.shtml). After aligning, duplicates were removed using the SAMBLASTER function^65^ and single nucleotide polymorphisms and inDels were called using the FreeBayes function.^66^ Errors introduced by random PCR or NGS were eliminated using the AccuraGen proprietary algorithm (AccuraGen, Shanghai, China). The following criteria were used for variant calling: 1) Different base call from the reference at the position of interest; 2) consistent difference between the tandem copies of the fragment sequence; 3) difference supported by more than one molecule; and 4) no germline or clonal hematopoietic mutations in the gDNA. The VAF was calculated as the number of variant reads divided by the total number of sequencing reads at the variant site. Sequencing of WBC samples with positive mutation scores allowed for the filtering of CHIP-induced somatic mutations. If a cfDNA variant was observed in the gDNA of WBCs and if the allelic fraction of the cfDNA variant was at least 10-fold higher than the allele frequency in WBCs for the cfDNA variant, the cfDNA variant was maintained for further analysis.

#### Whole-exon sequencing of tumor tissues

Tumor tissue gDNA was sequenced through whole exon sequencing by LC-Bio Technology Co., Ltd (Hangzhou, China). Briefly, gDNA was fragmented using a Covaris M220 Focused-ultrasonicator (Covaris) and then subjected to sequencing library construction. Exon capture was performed using the Human Exon 2.0 Plus kit (Twist Bioscience) following the protocol recommended by the vendor. The final libraries were sequenced for paired-end 150 bp using the Illumina NovaSeq 6000 sequencing system (Illumina).

Prior to alignment, low quality reads containing sequencing adaptors or nucleotides with q quality score less than 20 were removed using the fastp function.^67^ Burrows Wheeler Aligner (BWA)^68^ was used to align reads to the reference genome, hg19. In addition, in the first post-alignment processing step, the Picard tool (http://broadinstitute.github.io/picard/) was used to identify and mark duplicate reads from the BAM file. In the second post-alignment processing step, local read realignment was performed to correct for potential alignment errors around inDels. Then, base quality score recalibration was performed prior to variant calling to reduce systematic bias. Somatic SNVs and InDels were jointly called using Mutect2.^69^ ANNOVAR^70^ was used to add biological information to the variant set.

#### Protein biomarker analysis

The cobas platform (Roche Diagnostics, Indianapolis, IN, USA) was used to evaluate the levels of seven protein biomarkers previously shown to be significantly useful for cancer detection using plasma samples (n = 456).

#### Model development and evaluation

The samples in our cohort were randomly divided into the training (n = 295) and validation (n = 127) groups at a 7:3 ratio using the Scikit-learn (sklearn) function, and were used for modeling and model evaluation. The Sklearn function was also used to establish the LR models and predict probabilities following the development of the predictive models using the training dataset. We investigated all the possible combinations of eight variables (six proteins, age, and cfDNA concentration) with CA125+ctDNA using the training dataset, and mean values for accuracy, F1 score, and AUC with five-fold cross-validation were used to evaluate the performance of the different LR models. The model with the highest accuracy and F1 score, and the least number of variables was selected as the most performant model (Table S9).

The external validation cohort was selected from the CancerSEEK study and included 335 participants i.e., 54 patients with OC and 281 healthy controls. All patients with OC were included and healthy individuals were selected based on the criteria, 1) sex = female and 2) age ≥ 30 years.

We used predictive probability and disease status to draw receiver operator characteristic (ROC) curves for the models. Aside from CA125 and ROMA, the optimal cut-off values for the other models were obtained using the Youden index (sensitivity + specificity − 1) based on the ROC analysis. LR model fitting and threshold determination were performed only on training data and then applied to validation and external data. The performance of the models was assessed based on their sensitivity, specificity, PPV, NPV, and accuracy using the MedCalc platform (MedCalc Software, https://www.medcalc.org/calc/diagnostic_test.php).

The parameters for the ROMA model were calculated based on CA125 and HE4 plasma levels; the formula for calculating the predictive index (PI) of the ROMA model in the premenopausal population was PI = -12.0+2.38×LN(HE4) + 0.0626×LN(CA125), while that for calculating its PI in the postmenopausal population was PI = -8.09+1.04×LN(HE4) + 0.732×LN(CA125);ROMA = exp(PI)/[1+ exp(PI)]×100; the cut-off values for the ROMA model were 11.4 in the premenopausal population and 29.9 in the postmenopausal population. The cut-off value for CA125 was 35 U/mL.

### Quantification and statistical analysis

Statistical analysis and data visualization were performed in R (version 4.2.2; the R Foundation for Statistical Computing, Vienna, Austria; https://www.r-project.org/) using the “epiDisplay”, “ggVennDiagram”, “openxlsx”, “pROC”, “ggplot2”, “gmodels”, “DTComPair”, and “cowplot” functions. *p* < 0.05 was considered statistically significant (two-tailed test). Data randomization, variable selection, modeling, and performance evaluation of the models were performed using Python version 3.9 with the Scikit-learn (sklearn) version 1.3 package (https://scikit-learn.org/stable/).

## Supplemental information

Document S1. Figures S1–S6 and Tables S2, S3, S5, S6, S8, S10, S11, and S13–S16

Table S1. Subject characteristics of this study, related to Table 1

Table S4. Mutations identified in primary tumors of 32 ovarian cancer patients, related to Figure 2

Table S7. The external validation cohort from CancerSEEK, related to Figure 3

Table S9. Utilizing 5-fold cross-validation and sklearn to determine the optimal variable combination, related to Figure 4

Table S12. Comparison of models' performance metrics measured around 95%, 90% and 85% sensitivity in premenopausal and postmenopausal subjects, related to Table 2
