# Supplementary material for: Multi-analyte approach combining cfDNA sequencing and protein testing for early ovarian cancer detection
Source: iScience. 2025 May 8;28(6):112617. doi: 10.1016/j.isci.2025.112617 (PMC12152359; doi:10.1016/j.isci.2025.112617)
Supplement: Document S1. Figures S1–S6 and Tables S2, S3, S5, S6, S8, S10, S11, and S13–S16 [file mmc1.pdf]

iScience, Volume *28*

Supplemental information

Multi-analyte approach combining cfDNA sequencing and protein testing for early ovarian cancer detection

Fenfen Wang (王芬芬), Lingfang Wang (王玲芳), Ziyu Xing (幸子玉), Lingjia Lu (陆伶佳), Zhuoqun Lin (林卓群), Senmi Qian (钱森密), Tao Zhu (朱滔), Zhuyan Shao (邵株燕), Lingjun Zhao (赵玲军), Jie Dong (董婕), Fangfang Qian (钱芳芳), Yang Li (李阳), Xiaojing Chen (陈晓静), Siqi Yang (杨思琦), and Xiaodong Cheng (程晓东)

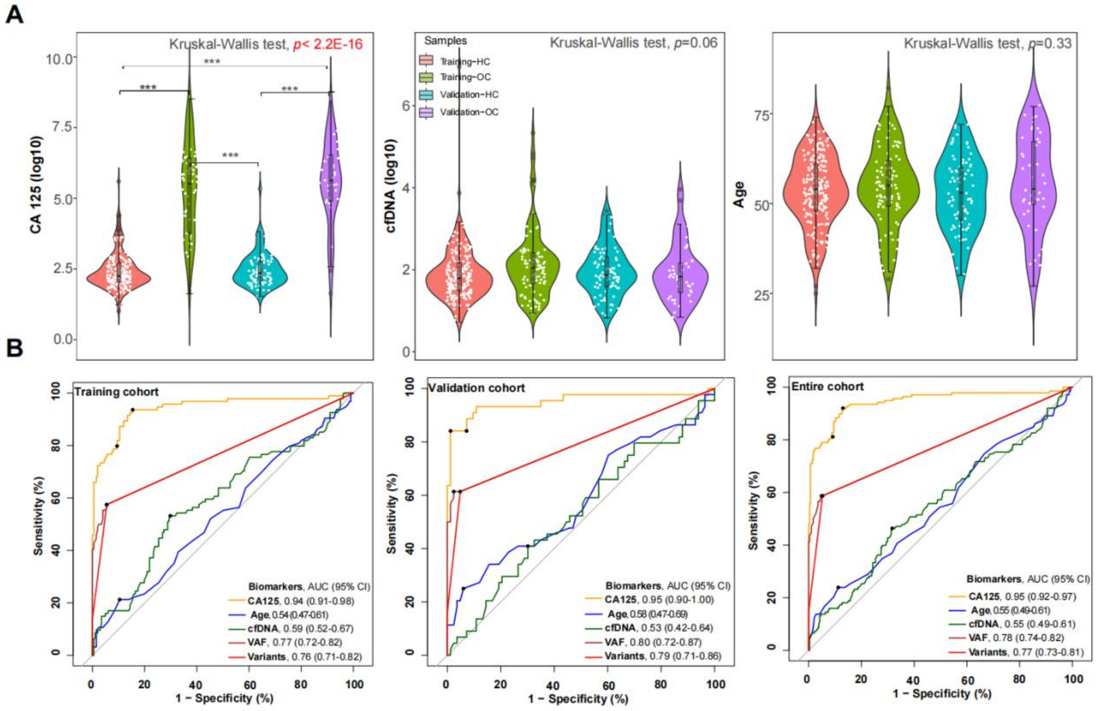

Biomarkers	P values of DeLong's test		
	Training cohort	Validation cohort	Entire cohort
CA125 versus Age	< 2.20E-16	5.35E-10	2.20E-16
CA125 versus cfDNA	< 2.20E-16	1.25E-11	2.20E-16
CA125 versus VAF	2.23E-09	2.36E-04	1.68E-12
CA125 versus Variants	3.56E-10	1.01E-04	1.24E-13
Age versus cfDNA	0.19	0.56	0.95
Age versus VAF	1.57E-07	2.48E-04	1.18E-10
Age versus Variants	3.50E-07	4.39E-04	4.55E-10
cfDNA versus VAF	1.16E-04	2.13E-04	2.02E-09
cfDNA versus Variants	2.09E-04	3.32E-04	6.63E-09
VAF versus Variants	8.13E-03	0.07	1.25E-03

Figure S1. Patients with ovarian cancer and healthy individuals were differentiated using different indicators. Related to Figure 3.

(A) Distribution of CA125 expression levels, cell-free DNA (cfDNA) concentrations, and age between patients with ovarian cancer and healthy individuals.

(B) Receiver operator characteristic (ROC) curves for various biomarkers in the different datasets. In total, 201 healthy individuals and 94 patients with OC were included in the training cohort, while 94 healthy individuals and 44 patients with OC were included in the validation cohort. The related p values were listed in the table.

cfDNA: cell-free DNA; HC: healthy control; OC: ovarian cancer; AUC: area under the curve; CI: confidence interval; VAF: variant allele frequency; “Variants” refers to the number of detected variants in each patient; ***, $p < 0.001$.

A

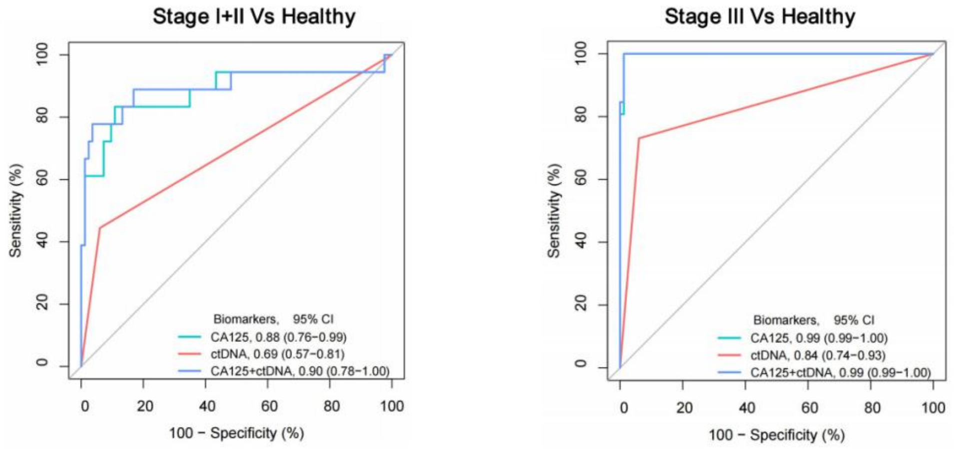

B

Cohorts	Statistics	Value(95% CI),%		
		CA125	ctDNA	CA125+ctDNA
Stage I+II Vs Healthy (n=101)	Sensitivity	89.2 (80.4-94.9)	94.0 (86.5-98.0)	96.4(89.8-99.2)
	Specificity	83.3 (58.6-96.4)	44.4 (21.5-69.2)	77.8(52.4-93.6)
	Accuracy	88.1 (80.2-93.7)	85.1 (76.7-91.4)	93.1(86.2-97.2)
Stage III Vs Healthy (n=109)	Sensitivity	98.8 (93.5-100.0)	94.0 (86.5-98.0)	98.8(93.5-100.0)
	Specificity	100.0 (86.8-100.0)	73.1 (52.2-88.4)	100.0(86.8-100.0)
	Accuracy	99.1 (95.0-100.0)	89.0 (81.6-94.2)	99.1(95.0-100.0)

Figure S2. Performance comparison among CA125 alone, ctDNA alone, and the combination model in stage I+II and stage III subgroup. Related to Figure 3.

(A) The OC patients in validation dataset were divided into stage I+II and stage III subgroups, receiver operator characteristic (ROC) curves for CA125 alone, ctDNA alone, and the combination test were established in the two subgroup. The *p* values in Stage I+II subgroup were 0.015, 0.47 and 0.003, in the compare among CA125 Vs ctDNA, CA125 Vs CA125+ctDNA, and ctDNA Vs CA125+ctDNA, respectively. The *p* values in Stage III subgroup were 0.0005, 0.48 and 0.0004, in the compare among CA125 Vs ctDNA, CA125 Vs CA125+ctDNA, and ctDNA Vs CA125+ctDNA, respectively.

(B) The sensitivity, specificity, and accuracy of CA125 alone, ctDNA alone, and CA125+ctDNA in the two subgroup.

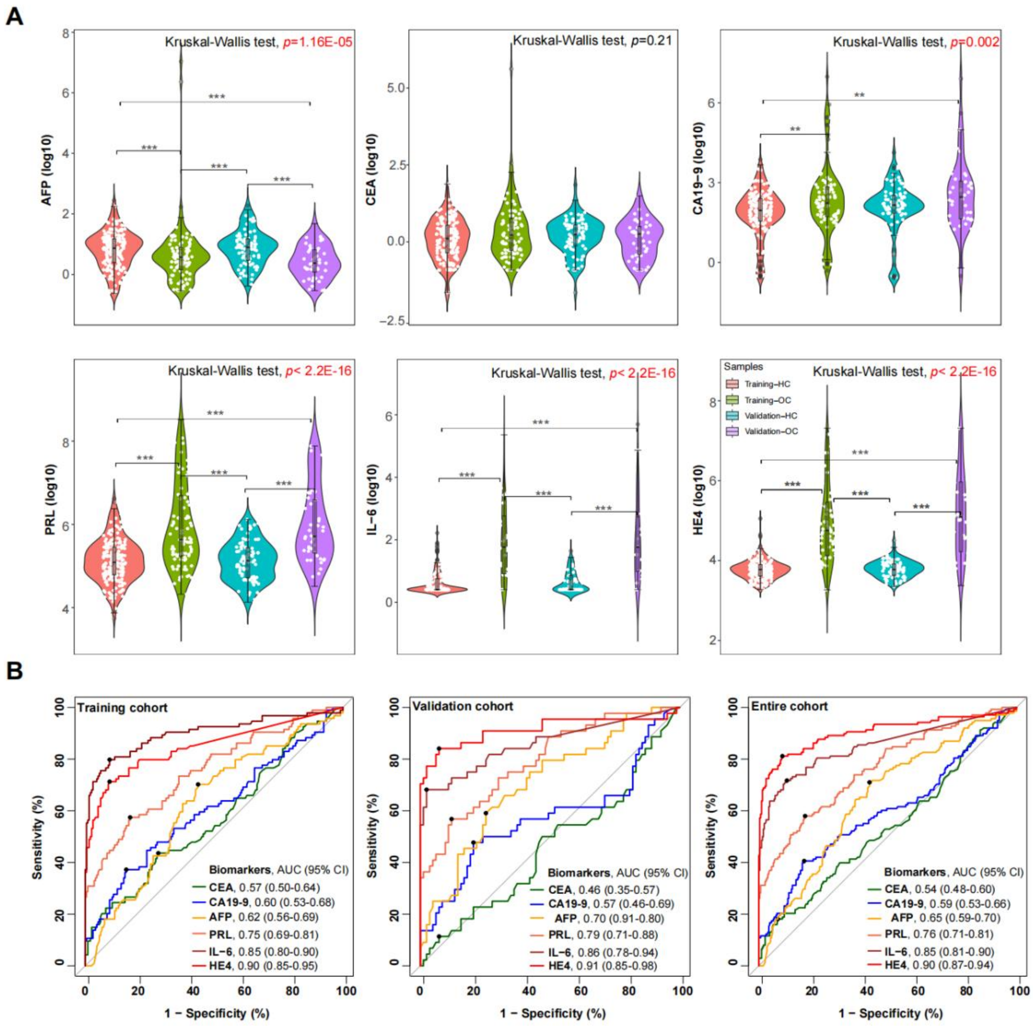

Biomarkers	P values of DeLong's test		
	Training cohort	Validation cohort	Entire cohort
CEA versus CA19-9	0.45	0.10	0.13
CEA versus AFP	0.29	8.51E-04	9.23E-03
CEA versus PRL	2.09E-04	2.45E-06	1.95E-08
CEA versus IL-6	6.19E-09	3.04E-08	3.20E-15
CEA verus HE4	2.92E-15	3.35E-12	< 2.2E-16
CA19-9 versus AFP	0.70	0.08	0.20
CA19-9 versus PRL	3.87E-03	1.14E-03	3.33E-05
CA19-9 versus IL-6	1.73E-07	7.66E-05	4.01E-11
CA19-9 versus HE4	8.88E-11	1.71E-07	< 2.2e-16
AFP versus PRL	7.55E-03	0.16	2.33E-03
AFP versus IL-6	3.52E-07	9.18E-03	8.50E-09
AFP versus HE4	3.13E-10	5.80E-04	6.53E-13
PRL versus IL-6	0.01	0.20	4.56E-03
PRL versus HE4	1.02E-04	0.013	3.74E-06
IL-6 versus HE4	0.10	0.17	0.04

Figure S3. Patients with ovarian cancer and healthy individuals were differentiated using protein biomarker. Related to Figure 4.

(A) Distribution of AFP, CEA, CA19-9, PRL, IL-6, and HE4 between patients with OC and healthy individuals.

(B) Receiver operator characteristic (ROC) curves for five protein biomarkers in the different datasets. In total, 201 healthy individuals and 94 patients with OC were included in the training cohort, while 83 healthy individuals and 44 patients with OC were included in the validation cohort. The related *p* values were listed in the table.

HC: healthy control; OC: ovarian cancer; AUC: area under the curve; CI: confidence interval; **, $p < 0.01$. ***, $p < 0.001$.

A

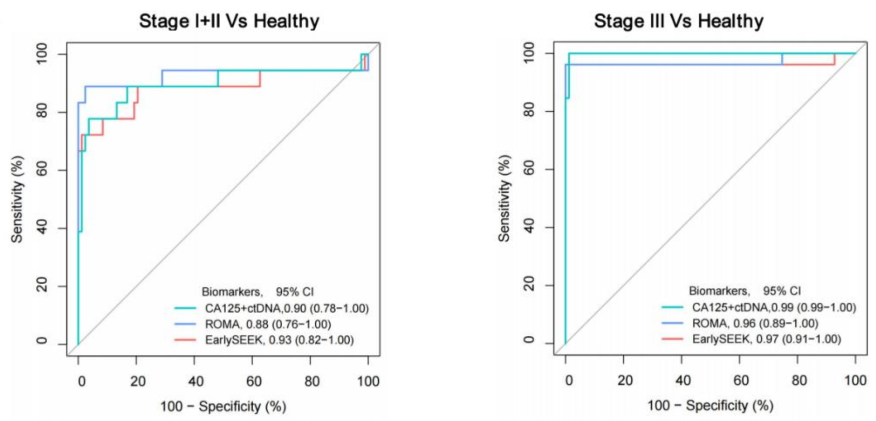

B

Cohorts	Statistics	Value(95% CI),%		
		CA125+ctDNA	ROMA	EarlySEEK
Stage I+II Vs Healthy (n=101)	Sensitivity	96.4(89.8-99.2)	98.8(93.5-100.07)	97.6(91.6-99.7)
	Specificity	77.8(52.4-93.6)	72.2(46.5-90.3)	88.9(65.3-98.6)
	Accuracy	93.1(86.2-97.2)	94.1(87.5-97.8)	96.0(90.2-98.9)
Stage III Vs Healthy (n=109)	Sensitivity	98.8(93.5-100.0)	100.0(95.7-100.0)	100.0(95.7-100.0)
	Specificity	100.0(86.8-100.0)	96.2(80.4-99.9)	96.2(80.4,99.9)
	Accuracy	99.1(95.0-100.0)	99.1(95.0-100.0)	99.1(95.0-100.0)

Figure S4. Performance comparison between the two combination tests. Related to Figure 4.

(A) The OC patients in validation dataset were divided into stage I+II and stage III subgroup, receiver operator characteristic (ROC) curves for the ROMA, CA125+ctDNA, and EarlySEEK tests were determined using the data from stage I+II and stage III subgroup. The *p* values in Stage I+II subgroup were 0.07 and 0.28, in the compare among EarlySEEK Vs ROMA and EarlySEEK Vs CA125+ctDNA, respectively. The *p* values in Stage III subgroup were 0.89 and 0.35, in the compare among EarlySEEK Vs ROMA and EarlySEEK Vs CA125+ctDNA, respectively.

(B) The sensitivity, specificity, and accuracy of CA125+ctDNA, ROMA and EarlySEEK in the two subgroups.

A

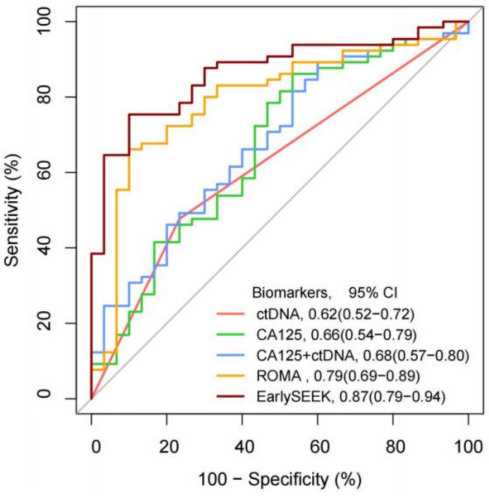

B

Biomarkers	Statistics	Value(95%CI),%	Versus CA125	Versus ROMA
CA125	Sensitivity	46.7(28.3, 65.7)	-	<0.001
	Specificity	86.2(75.3, 93.5)	-	0.014
	PPV	60.9 (38.5, 80.3)	-	0.837
	NPV	77.8(66.4, 86.7)	-	0.045
	Accuracy	73.7(63.6, 82.2)	-	1.000
CA125+ ctDNA	Sensitivity	40.0(22.7, 59.4)	0.795	<0.001
	Specificity	89.2 (79.1, 95.6)	0.790	0.003
	PPV	63.2 (38.4, 83.7)	1.000	0.742
	NPV	76.3 (65.2, 85.3)	0.987	0.029
	Accuracy	73.7(63.6, 82.2)	1.000	1.000
ROMA	Sensitivity	90.0 (73.5, 97.9)	<0.001	-
	Specificity	66.2 (53.4, 77.4)	0.014	-
	PPV	55.1 (40.2, 69.3)	0.837	-
	NPV	93.5(82.1, 98.6)	0.045	-
	Accuracy	73.7 (63.6, 82.2)	1.000	-
EarlySEEK	Sensitivity	90.0 (73.5, 97.9)	<0.001	1.000
	Specificity	75.4 (63.1, 85.2)	0.182	0.335
	PPV	62.8(46.7, 77.0)	1.000	0.593
	NPV	94.2(84.1, 98.8)	0.024	1.000
	Accuracy	80.0(70.5, 87.5)	0.390	0.390

Figure S5. Performance comparison between different biomarkers in distinguishing benign from early-stage ovarian tumors. Related to Figure 5.

(A) ROC curves for distinguishing ovarian tumors in the early stages (stage I, II). Different colors represent different variables or variable combinations; 30 benign tumors and 65 patients with stage I or stage II OC were included.The *p* values were < 0.001, 0.0006, 0.0003 and 0.08, in the compare among EarlySEEK Vs ctDNA, EarlySEEK Vs CA125, EarlySEEK Vs CA125+ctDNA and EarlySEEK Vs ROMA, respectively.

(B) Comparison of sensitivity, specificity, PPV, NPV and accuracy between various biomarkers. ctDNA: circulating tumor DNA; PPV: positive predictive value; NPV: negative predictive value; ROC: receiver operator characteristics.

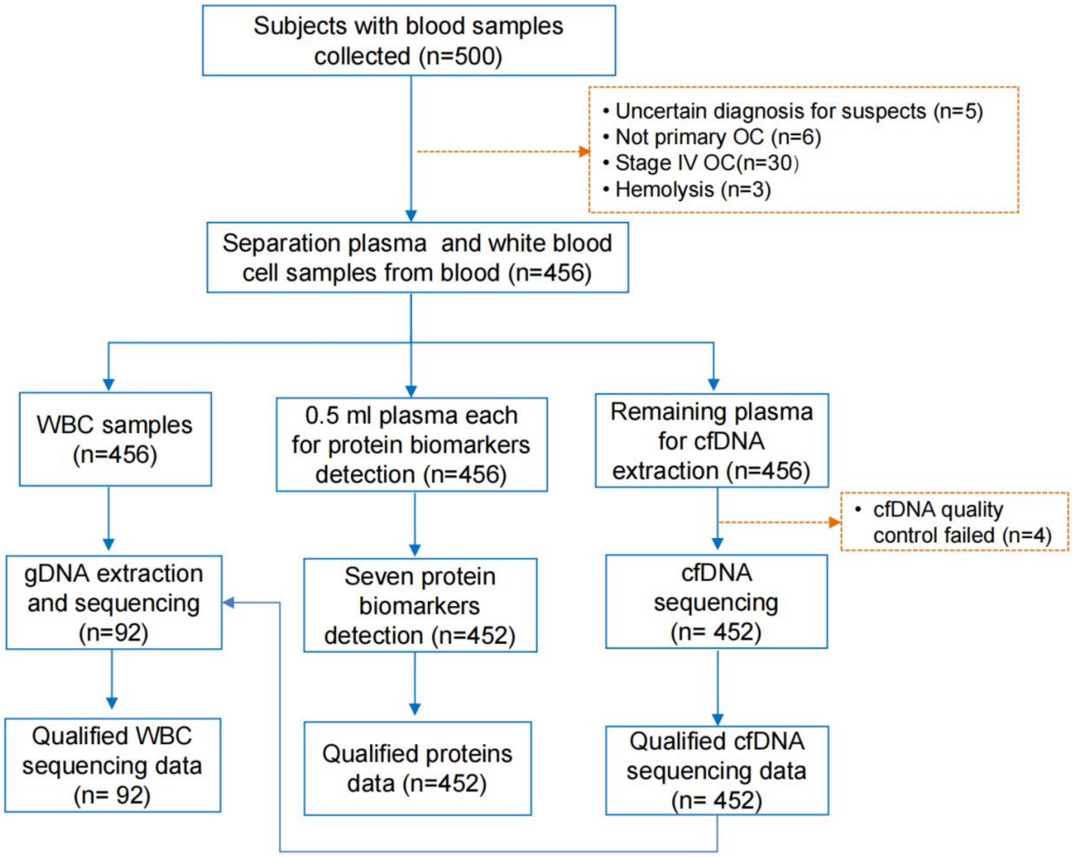

Figure S6. Workflow of the quality control process. Related to STAR Methods.

A total of seven patients were excluded from this quality control process, including three cases were removed in the sample collection process due to hemolysis; four subjects were eliminated for unqualified cfDNA samples in the DNA extraction process, in which three cfDNA samples were severely contaminated with gDNA (> 50%), one sample had insufficient cfDNA yield (< 5 ng).

Table S2. Comparison of histological subtypes among different stages of ovarian cancer. Related to Table 1.

Histological subtypes	Stages [n (percentage)]			P value (Fisher's exact test)
	I（N=38）	II （N=27）	III （N=73）	
**Non-EOC**	10 (26.32)	3 (11.11)	4 (5.48)	0.01
**EOC**	28 (73.68)	24 (88.89)	69 (94.52)	
Clear cell carcinoma	7 (18.42)	2 (7.41)	6 (8.22)	< 0.001
Endometrioid carcinoma	4 (10.53)	1 (3.7)	3 (4.11)	
High-grade serous carcinoma	12 (31.58)	19 (70.37)	60 (82.19)	
Low-grade serous carcinoma	1 (2.63)	1 (3.7)	0 (0)	
Mucinous carcinoma	4 (10.53)	1 (3.7)	0 (0)	

Table S3. ctDNA detection by histological types and stages.Related to Figure 2.

Gene	Mutation [N(%)]			P value
	OC (N=138)	EOC (N=121)	Non-EOC (N=17)	
18 genes	81 (58.7)	74 (61.2)	7 (41.2)	0.117
TP53	58 (42)	54 (44.6)	4 (23.5)	0.1
PIK3CA	10 (7.2)	9 (7.4)	1 (5.9)	0.644
APC	9(6.5)	8 (6.6)	1 (5.9)	0.694
CDKN2A	4 (2.9)	2 (1.7)	2 (11.8)	0.074
PPP2R1A	4 (2.9)	4 (3.3)	0 (0)	0.587

EOC	Detected	Total	Detection rate
serous carcinoma	63	93	67.7%
clear cell carcinoma	5	15	33.3%
endometrioid carcinoma	2	8	25.0%
mucinous carcinoma	4	5	80.0%

Stages	P value		
	Stage I	Stage II	Stage III
Stage I	/	0.038	0.001
Stage II	0.038	/	0.602
Stage III	0.001	0.602	/

OC, ovarian cancer; EOC, epithelial ovarian cancer.

Table S5. cfDNA features among different groups.Related to Figure 2.

**Detection rate of ctDNA variants**			
	Ovarian cancer	Non-cancer control	P value
ctDNA variants detected	81	22	p<0.001
No ctDNA variants detected	57	292	
Total	138	314	
	Ovarian cancer	Healthy individuals	
ctDNA variants detected	81	15	p<0.001
No ctDNA variants detected	57	269	
Total	138	284	
	Ovarian cancer	Benign tumor	
ctDNA variants detected	81	7	p<0.001
No ctDNA variants detected	57	23	
Total	138	30	
	Benign tumor	Healthy individuals	
ctDNA variants detected	7	15	p<0.001
No ctDNA variants detected	23	269	
Total	30	284	

**Distribution of mean variants allele frequency**				
Group	Ovarian cancer	Healthy individuals	Benign tumor	P value
Median	0.60%	0.14%	0.19%	< 0.001
Q1	0.24%	0.08%	0.17%	
Q3	5.08%	0.21%	0.21%	
Groups	P value			
healthy-benign	0.508			
healthy-OC	0			
benign-OC	0.009			

**cfDNA concentration**				
cfDNA concentration	Ovarian cancer	Healthy individuals	Benign tumor	P value
Median	7.22	6.24	6.14	0.163
Q1	4.86	4.52	3.54	
Q3	10.74	9.05	9.74	

cfDNA, cell-free DNA, ctDNA, circulating tumor DNA; Q1, the first quartile; Q3, the third quartile.

Table S6. Performance comparison and statistical analysis of different biomarkers.Related to Figure 3.

Cohorts	Statistics	Value (95 CI), %			P values		
		CA125	ctDNA	CA125+ctDNA	CA125 vs. ctDNA	CA125 vs. CA125+ctDNA	ctDNA vs. CA125+ctDNA
Training	Sensitivity	78.7 (69.1 - 86.5)	57.5 (46.8 - 67.6)	84.0 (75.1 - 90.8)	0.0004	0.06	<0.0001
	Specificity	90.6 (85.6 - 94.2)	94.5 (90.4 - 97.2)	97.0 (93.6 - 98.9)	0.13	0.0008	0.17
	PPV	79.6 (71.5 - 85.8)	83.1 (72.9 - 90.0)	92.9 (85.6 - 96.7)	0.53	0.0006	0.04
	NPV	90.1 (86.0 - 93.1)	82.6 (78.9 - 85.8)	92.9 (89.1 - 95.4)	0.0009	0.02	<0.0001
	Accuracy	86.8 (82.4 - 90.4)	82.7 (77.9 - 86.9)	92.9 (89.3 - 95.5)	0.12	0.0001	<0.0001
Validation	Sensitivity	84.1 (69.9 - 93.4)	61.4 (45.5 - 75.6)	90.9 (78.3 - 97.5)	0.01	0.08	0.0003
	Specificity	92.8 (84.9 - 97.3)	95.2 (88.1 - 98.7)	97.6 (91.6 - 99.7)	0.53	0.10	0.32
	PPV	86.1 (73.8 - 93.1)	87.1 (71.6 - 94.8)	95.2 (83.5 - 98.8)	0.89	0.09	0.16
	NPV	91.7 (84.8 - 95.6)	82.3 (76.2 - 87.1)	95.3 (88.8 - 98.1)	0.02	0.07	0.0003
	Accuracy	89.8 (83.1 - 94.4)	83.5 (75.8 - 89.5)	95.3 (90.0 - 98.3)	0.12	0.02	0.0003
Entire	Sensitivity	80.4 (72.8 - 86.7)	58.7 (50.0 - 67.0)	84.8 (77.7 - 90.3)	<0.0001	0.06	<0.0001
	Specificity	91.2 (87.3 - 94.2)	95.1 (91.9 - 97.3)	97.5 (95.0 - 99.0)	0.07	<0.0001	0.09
	PPV	81.6 (75.2 - 86.7)	85.3 (77.3 - 90.8)	94.4 (88.9 - 97.2)	0.42	<0.0001	0.02
	NPV	90.6 (87.2 - 93.1)	82.6 (79.5 - 85.3)	93.0 (89.9 - 95.1)	<0.0001	0.02	<0.0001
	Accuracy	87.7 (84.2 - 90.7)	83.2 (79.3 - 86.6)	93.4 (90.6 - 95.6)	0.04	<0.0001	<0.0001

ctDNA, circulating tumor DNA; PPV, positive predictive value; NPV, negative predictive value, vs., versus.

Table S8. Univariate and multivariate analysis by logistic regression. Related to Figure 4.

Variables	OR	95% CI	P value	OR	95%CI	P value
IL-6	2.04	1.65 - 2.52	<0.0001	1.40	1.03 - 1.91	0.031
PRL	1.01	1 - 1.01	<0.0001	1.01	1 - 1.01	0.027
ctDNA VAF	593499.65	4417.28 - 79741813.367	<0.0001	1913847.85	205.38 - 17834499123.88	0.002
CA125	1.03	1.02 - 1.05	<0.0001	1.02	1.01 - 1.03	0.001
HE4	1.09	1.06 - 1.12	<0.0001	1.09	1.04 - 1.13	<0.0001
Number of ctDNA variants	21.98	10.35 - 46.7	<0.0001	-	-	-
CA 19-9	1.03	1.01 - 1.06	0.006	-	-	-
CEA	1.34	1.09 - 1.64	0.006	-	-	-
Age	1.03	1.01 - 1.06	0.011	-	-	-
AFP	1.01	0.99 - 1.02	0.333	-	-	-
cfDNA	1.00	0.997 - 1	0.846	-	-	-

IL-6, interleukin 6; PRL, prolactin; ctDNA, circulating tumor DNA; VAF, variant allele frequency; CA125, cancer antigen 125; HE4 human epididymis protein 4; CA19-9, cancer antigen 19-9; CEA, carcinoembryonic antigen; AFP, Alpha-Fetoprotein; cfDNA, cell-free DNA; CI, confidence interval; OR, odds ratio.

Table S10. Performance comparison and statistical analysis of three combined models.Related to Figure 4.

Cohorts	Statistics	Value (95 CI), %			P values		
		CA125+ctDNA	ROMA	EarlySEEK	CA125+ctDNA vs. ROMA	CA125+ctDNA vs. EarlySEEK	ROMA vs. EarlySEEK
Training	Sensitivity	84.0 (75.1 - 90.8)	75.5 (65.6 - 83.8)	90.4 (82.6 - 95.5)	/	/	4.65
	Specificity	97.0 (93.6 - 98.9)	97.5 (94.3- 99.2)	98.5 (95.7 - 99.7)	/	/	0.32
	PPV	92.9 (85.6 - 96.7)	93.4 (85.6 - 97.1)	96.5 (90.2 - 98.9)	0.88	0.22	0.20
	NPV	92.9 (89.1 - 95.4)	89.5 (85.7 - 92.4)	95.7 (92.2 - 97.6)	0.01	0.10	4.29
	Accuracy	92.9 (89.3 - 95.5)	90.5 (86.6 - 93.6)	95.9 (93.0 - 97.9)	0.02	5.34	3.47
Validation	Sensitivity	90.9 (78.3 - 97.5)	81.8 (67.3 - 91.8)	93.2 (81.3 - 98.6)	/	/	0.03
	Specificity	97.6 (91.6 - 99.7)	100.0 (95.7 - 100.0)	100 (95.7 - 100.0)	/	/	1.00
	PPV	95.2 (83.5 - 98.8)	100.0 (90.3 - 100.0)	100 (91.4 - 100.0)	0.16	0.16	1.00
	NPV	95.3 (88.8 - 98.1)	91.2 (84.7 - 95.1)	96.5 (90.3 - 98.8)	0.11	0.53	0.03
	Accuracy	95.3 (90.0 - 98.3)	93.7 (88.0 - 97.2)	97.6 (93.3 - 99.5)	2.75	1.62	2.21
Entire	Sensitivity	84.8 (77.7 - 90.3)	77.5 (69.7 - 84.2)	91.3 (85.3 - 95.4)	/	/	3.38
	Specificity	97.5 (95.0 - 99.0)	98.2 (95.9 - 99.4)	98.6 (96.4 - 99.6)	/	/	0.65
	PPV	94.4 (88.9 - 97.2)	95.5 (89.9 - 98.1)	96.9 (92.2 - 98.8)	0.60	0.21	0.45
	NPV	93 (89.9 - 95.1)	90.0 (86.8 - 92.5)	95.9 (93.1 - 97.6)	8.72	0.03	3.28
	Accuracy	93.4 (90.6 - 95.6)	91.4 (88.4 - 94.0)	96.2 (93.9 - 97.8)	6.07	1.26	8.77

CI, confidence interval; ctDNA, circulating tumor DNA; PPV, positive predictive value; NPV, negative predictive value, vs., versus.

Table S11.: The cinical information and predictive results of the ovarian cancer patients missed by CA125.Related to Figure 4.

Patient	Cancer	Stage	Age	Histological subtyoe	CA125 level (U/ml) (0-35.00)	HE4 (pmol/L) 0-70 (premenopausal) 0-140 (postmenopausal)	Predictive probability				Evaluation at 95% sensitivity				Evaluation at 98% sensitivity			
							ctDNA	ctDNA+ CA125	ROMA	Early SEEK	ctDNA	ctDNA+ CA125	ROMA	Early SEEK	ctDNA	ctDNA+ CA125	ROMA	Early SEEK
OCED 007	EOC	I	58	HGSC	24.1	57.93	0.180	0.111	17.67	0.999	FN	FN	TP	TP	FN	FN	FN	TP
OCED 013	EOC	I	57	CCC	34.79	40.84	0.180	0.153	16.33	1.000	FN	FN	TP	TP	FN	FN	FN	TP
OCED 020	EOC	I	56	CCC	15.35	96.59	0.180	0.084	20.79	0.407	FN	FN	TP	TP	FN	FN	FN	TP
OCED 040	EOC	I	66	HGSC	12.61	53.35	0.180	0.077	10.92	1.000	FN	FN	FN	TP	FN	FN	FN	TP
OCED 042	Non-EOC	I	66	Non-EOC	29.58	65.74	0.180	0.131	22.14	1.000	FN	FN	TP	TP	FN	FN	TP	TP
OCED 051	EOC	I	50	MC	13.24	59.87	0.605	0.271	12.53	0.997	TP	FN	FN	TP	FN	FN	FN	TP
OCED 074	EOC	I	71	LGSC	32.19	111.4	0.180	0.142	34.36	1.000	FN	FN	TP	TP	FN	FN	TP	TP
OCED 076	Non-EOC	I	52	Non-EOC	5.07	33.19	0.180	0.060	3.70	0.022	FN	FN	FN	FN	FN	FN	FN	FN
OCED 089	Non-EOC	I	68	Non-EOC	33.11	102.4	1.000	1.000	32.87	1.000	TP	TP	TP	TP	TP	TP	TP	TP
OCED 101	EOC	I	47	HGSC	20.07	56.64	0.180	0.098	9.93	1.000	FN	FN	FN	TP	FN	FN	FN	TP

OCED 105	EOC	I	50	CCC	23.27	70.75	0.180	0.108	20.48	0.135	FN	FN	TP	FN	FN	FN	FN	FN
OCED 129	EOC	I	35	MC	9.45	26.22	0.742	0.321	1.65	0.120	TP	TP	FN	FN	TP	FN	FN	FN
OCED 137	EOC	I	56	HGSC	28.94	600	0.180	0.129	73.62	1.000	FN	FN	TP	TP	FN	FN	TP	TP
OCED 138	EOC	I	67	HGSC	30.03	50.62	0.627	0.414	17.97	0.209	TP	TP	TP	TP	FN	TP	FN	FN
OCED 144	EOC	II	70	HGSC	30.81	87.46	0.623	0.418	28.28	0.417	TP	TP	TP	TP	FN	TP	TP	TP
OCED 149	EOC	I	49	HGSC	19.54	45.76	0.697	0.371	6.21	0.315	TP	TP	FN	TP	TP	TP	FN	FN
OCED 151	Non-EOC	I	33	Non-EOC	5.14	29.17	0.180	0.060	2.04	0.008	FN	FN	FN	FN	FN	FN	FN	FN
OCED 166	EOC	II	74	EC	11.51	51.2	0.180	0.074	9.90	0.992	FN	FN	FN	TP	FN	FN	FN	TP
OCED 179	Non-EOC	I	29	Non-EOC	19.01	28.57	1.000	1.000	2.11	1.000	TP	TP	FN	TP	TP	TP	FN	TP
OCED 185	EOC	I	52	MC	6.18	38.8	0.180	0.062	4.96	0.014	FN	FN	FN	FN	FN	FN	FN	FN
OCED 186	EOC	I	54	HGSC	14.18	49.15	0.180	0.081	10.93	0.077	FN	FN	FN	FN	FN	FN	FN	FN
OCED 213	EOC	I	34	CCC	21.89	44.7	0.180	0.103	5.94	0.049	FN	FN	FN	FN	FN	FN	FN	FN
OCED 233	EOC	II	57	HGSC	26.61	100	0.706	0.437	28.93	1.000	TP	TP	TP	TP	TP	TP	TP	TP

OCED 240	Non-EOC	II	67	Non-EOC	26.63	57.99	0.926	0.739	18.77	1.000	TP	TP	TP	TP	TP	TP	FN	TP
OCED 242	Non-EOC	I	31	Non-EOC	21.69	47.85	0.180	0.103	6.90	0.213	FN	FN	FN	TP	FN	FN	FN	FN
OCED 244	Non-EOC	I	31	Non-EOC	34.07	50.3	0.180	0.150	7.91	0.669	FN	FN	FN	TP	FN	FN	FN	TP
OCED 245	Non-EOC	II	34	Non-EOC	34.71	39.74	0.180	0.153	4.68	1.000	FN	FN	FN	TP	FN	FN	FN	TP

EOC, epithelial ovarian cancer; CCC, clear cell carcinoma;MC,mucinous carcinoma ;HGSC, high-grade serous carcinoma; LGSC,low-grade serous carcinoma; TP, true positive; FP, false positive; TN, true negative; FN, false negative

Table S13. Comparison of age and models' performance between benign and malignant ovarian tumors. Related to Figure 5.

Groups	Variables	Benign tumor	Ovarian cancer	Statistical tests	P values
Overall	Number of patients	30	138		
	Mean (SD)	48.6 (13.1)	55.1 (11.7)	t-test	0.008
Age< 50	Total	15	35		
	Median (IQR)	41 (31.5,45)	40 (34.5,46.5)	Mann–Whitney U test	0.408
Age≥50	Number	15	103		
	Median (IQR)	56 (52,65)	58 (52.5,67)	Mann–Whitney U test	0.435

Variables	AUC (95% CI)		P values
	Age<50	Age≥50	
ctDNA	0.66 (0.54-0.78)	0.65 (0.52-0.78)	0.89
CA125	0.71 (0.55-0.86)	0.85 (0.73-0.96)	0.17
ctDNA+CA125	0.75 (0.60-0.90)	0.83 (0.72-0.95)	0.39
ROMA	0.84 (0.73-0.95)	0.87 (0.77-0.98)	0.65
EarlySEEK	0.95 (0.90-1.00)	0.92 (0.86-0.98)	0.42

Variables	P values of AUC comparison	
	Age<50	Age≥50
ctDNA vs. CA125	0.64	0.015
ctDNA vs. ctDNA+CA125	0.19	0.00778
ctDNA vs. ROMA	0.022	0.0036
ctDNA vs. EarlySEEK	<0.0001	<0.0001
CA125 vs. ctDNA+CA125	0.35	0.39
CA125 vs. ROMA	0.04	0.06
CA125 vs. EarlySEEK	<0.0001	0.13
ctDNA+CA125 vs. ROMA	0.19	0.02
ctDNA+CA125 vs. EarlySEEK	0.004	0.05
ROMA vs. EarlySEEK	0.01	0.24

CI, confidence interval; AUC, area under the curve; ctDNA, circulating tumor DNA; PPV, positive predictive value; NPV, negative predictive value, vs., versus.

Table S14. Comparison of the biomarkers utilized in different studies. Related to Discussion.

Biomarkers	PapSEEK	Maritschnegg's study	CanceSEEK	This study
**Gene panel**				
AKT1	√	√	√	√
APC	√	√	√	√
BRAF	√	√	√	√
CDKN2A	√	√	√	√
CTNNB1	√	√	√	√
EGFR	√	√	√	√
FBXW7	√	√	√	√
FGFR2	√	√	√	√
KRAS	√	√	√	√
NRAS	√	√	√	√
PIK3CA	√	√	√	√
PPP2R1A	√	√	√	√
PTEN	√	√	√	√
TP53	√	√	√	√
PIK3R1	√	√	×	×
POLE	√	√	×	×
RNF43	√	×	×	√
MAPK1	√	×	×	×
GNAS	×	×	√	√
HRAS	×	×	√	√
TOP2A	×	×	×	√
**Protein biomarkers**				
CA-125	/	/	√	√
CA19-9	/	/	√	√
PRL	/	/	√	√
CEA	/	/	√	×
MPO	/	/	√	×
OPN	/	/	√	×
HGF	/	/	√	×
TIMP-1	/	/	√	×
HE4	/	/	×	√
IL-6	/	/	×	√

CA125, cancer antigen 125; CA19-9, cancer antigen 19-9; PRL, prolactin; CEA, carcinoembryonic antigen; MPO, myeloperoxidase; OPN, osteopontin; HGF, hepatocyte growth factor; TIMP-1, tissue inhibitor of metallopro-teinases 1; HE4 human epididymis protein 4; IL-6, interleukin 6.

Table S15: Participant inclusion and exclusion criteria. Related to STAR Methods.

Cohort	Inclusion Criteria	Exclusion Criteria
All	● Age ≥18 years old women<br>● Plasma volume ≥3 mL or cell-free DNA≥ 10 ng	●Pregnant women (by self-report);<br>● Febrile illness or acute or severe disease that caused an inflammatory reaction or took antibiotics within two weeks before the blood draw;<br>●Participating in other clinical trials (including bowel preparation, anesthesia, etc.) that required taking drugs within two months before the blood draw;<br>● Recipient of organ transplant or prior non-autologous bone marrow or stem cell transplant or blood transfusion
Cancer	● Diagnosed with primary ovarian cancer	●Had received antitumor therapy prior to the blood collection, including chemotherapy, radiation therapy, immunotherapy, targeted therapy and cryotherapy.<br>●Tumors originating from other sites or multiple cancer types<br>● Stage IV ovarian cancer
Benign tumor	● Diagnosed with benign ovarian tumor	● Known or prior diagnosis of cancer
Healthy	/	● Known or prior diagnosis of cancer<br>●Infectious blood disease (except for hepatitis B virus or hepatitis C virus infection)

Table S16. Target regions of 18 gene sequencing panel. Related to STAR Methods.

Gene	Chr	Start	End	Length
NRAS	chr1	115252045	115252409	365
NRAS	chr1	115256378	115256589	212
NRAS	chr1	115258646	115258851	206
PTEN	chr10	89692664	89693103	440
FGFR2	chr10	123279507	123279812	306
HRAS	chr11	534131	534425	295
KRAS	chr12	25378412	25378779	368
KRAS	chr12	25380114	25380318	205
KRAS	chr12	25398128	25398373	246
AKT1	chr14	105246451	105246665	215
TP53	chr17	7572827	7573143	317
TP53	chr17	7573810	7574148	339
TP53	chr17	7576762	7577728	967
TP53	chr17	7578037	7578709	673
TP53	chr17	7579157	7579980	824
TOP2A	chr17	38569016	38569228	213
RNF43	chr17	56435025	56435292	268
PPP2R1A	chr19	52715820	52716049	230
GNAS	chr20	57484280	57484484	205
CTNNB1	chr3	41265963	41266213	251
PIK3CA	chr3	178916716	178917014	299
PIK3CA	chr3	178921400	178921686	287
PIK3CA	chr3	178935955	178936221	267
PIK3CA	chr3	178951936	178952216	281
FBXW7	chr4	153247237	153247451	215
FBXW7	chr4	153249254	153249508	255
FBXW7	chr4	153251757	153252033	277
APC	chr5	112116458	112116669	212
APC	chr5	112173849	112174055	207
APC	chr5	112174486	112174753	268
APC	chr5	112175057	112175794	738
EGFR	chr7	55227779	55228013	235
EGFR	chr7	55259369	55259675	307
BRAF	chr7	140453016	140453251	236
CDKN2A	chr9	21970951	21971358	408
